# The mechanism (Phe362Tyr mutation) behind resistance in *Lepeophtheirus salmonis* pre-dates organophosphate use in salmon farming

**DOI:** 10.1038/s41598-017-12384-6

**Published:** 2017-09-27

**Authors:** Kiranpreet Kaur, Francois Besnier, Kevin A. Glover, Frank Nilsen, Vidar Teis Aspehaug, Helene Børretzen Fjørtoft, Tor Einar Horsberg

**Affiliations:** 1 NMBU School of Veterinary Science, Sea Lice Research Centre, PO Box 8146 Dep., NO-0033 Oslo, Norway; 2 Institute of Marine Research, Nordnes, Bergen, Norway; 3 Department of Biology, University of Bergen, Sea Lice Research Centre, Bergen, Norway; 4 PatoGen Analyse AS, PO Box 1527, 6025 Ålesund, Norway; 5 Department of Biological Sciences in Ålesund, Norwegian University of Science and Technology, Ålesund, Norway

## Abstract

The salmon louse is an ectoparasitic copepod of salmonids in the marine environment, and represents a global challenge to salmon aquaculture. A major issue is the reliance of the industry on a limited number of chemicals to delouse salmonids on farms, and the high levels of resistance that lice have developed to all of these agents. However, for most of these chemicals, resistance and dispersal mechanisms are unknown. We recently demonstrated that the Phe362Tyr mutation is the primary cause of organophosphate resistance in lice collected on Norwegian farms. In the present study, we genotyped >2000 lice collected throughout the entire North Atlantic in the period 1998–2016, using Phe362Tyr and nine tightly linked SNPs. Our results showed that the Phe362Tyr mutation is strongly linked to lice survival following chemical treatment on farms located throughout the North Atlantic, demonstrating for the first time, that this mutation represents the primary mechanism for organophosphate resistance in salmon lice across the North Atlantic. Additionally, we observed multiple and diverse high frequency haplotypes linked with the allele conveying resistance to organophosphate. We, therefore, conclude that Phe362Tyr is not a *de novo* mutation, but probably existed in salmon lice before the introduction of organophosphates in commercial aquaculture.

## Introduction

*Lepeophtherius salmonis*, commonly known as the salmon louse, is an ectoparasitic copepod that infects salmonids in the marine environment. Lice feed on mucus, epidermal tissue and blood, and constitute a significant disease challenge during the marine phase of the life cycle for salmonids in both the Atlantic and Pacific oceans in its two sub-species forms^1^. The effects of salmon lice infections on host fish include stress, reduced growth and suppression of the immune system^2,3^. At higher infection rates, skin lesions and secondary infections may occur, and if left untreated, will ultimately lead to mortality^4^.

The salmon louse represents a major challenge to global Atlantic salmon (*Salmo salar*) farming, leading to huge economic losses, mainly attributed to the treatment costs; along with negative impacts on growth rates, product downgrading^5,6^, and in extreme circumstances emergency slaughter resulting from infections. In addition to direct challenges related to profitability and animal welfare, high salmon lice numbers have been observed on wild salmonids in farming-dense regions^7^, raising questions about transfer of infection from farmed to wild fish, and environmental sustainability of current rearing-practices^8^. Consequently, effective control of the salmon louse in salmonid aquaculture is essential in order to ensure the health and welfare of farmed fish, and limit the potential impacts of aquaculture on wild fish populations^9^.

A wide range of integrated pest management strategies have been developed to control salmon lice infections on farmed salmon. These include cleaner fish^10^, thermolicer^11^ and alternative production forms limiting the settlement of lice^12^. In addition, the industry is investigating the potential of breeding for increased resistance in host-fish^13,14^, and attempting to develop vaccines^15,16^. Despite this suite of control strategies, the industry is ultimately reliant on a limited number of chemical therapeutants applied as bath or in-feed treatments to control infections^17^. Nevertheless, the availability of a mere handful of licensed chemicals and their long-term and extensive use has resulted in high level of resistance in salmon lice to most of them^18–23^. This situation has become acute since no new chemicals have been introduced for lice control since 1999 when emamectin benzoate (SLICE) was first introduced to the Norwegian market^24^. A better understanding of the ways in which chemical resistance develops and disperses in lice is essential for us to manage new delousing agents when they emerge, and prolong the lives of existing chemicals.

Organophosphates (OPs) were the first chemicals introduced to delouse salmon in commercial aquaculture in Europe in the late 1970s. Since introduction, they have been extensively used in Norway and the UK, and until the mid-1990s, OPs were the major chemical used to control lice infestations on commercial farms^5,23^. In the early 1990s, decreased sensitivity and resistance started emerging in fish-farms in the UK and Norway, developing into a major issue in the late 1990s^20,23^. The increase in treatment failures due to resistant parasites led to the termination of OPs usage in Norway in 1999. After nearly a decade of non-use, an OP (azamethiphos) was once again re-introduced to delouse farmed salmonids in 2008^25^, due to the development of lice resistance to the other chemicals that were used extensively during this period^21–23^. However, despite nearly a decade of low or non-use, reports of reduced efficacy of azamethiphos treatments emerged from the field already in 2009, merely a year following its re-introduction. In 2013 and 2014, a national surveillance program, using bioassays to test for resistance, revealed a widespread distribution of azamethiphos resistance on Norwegian fish farms^26,27^.

OPs are inhibitors of the enzyme acetylcholinesterase (AChE). Acetylcholinesterase (AChE) is a serine hydrolase whose primary function is to terminate synaptic transmission at cholinergic synapses of both vertebrates and invertebrates by hydrolyzing the neurotransmitter acetylcholine (ACh)^28^. Recently, we demonstrated that a single mutation (Phe362Tyr) in the gene coding for acetylcholinesterase (AChE), represented the primary mechanism behind resistance in salmon lice towards azamethiphos^29^. This mutation was shown to affect the access and binding of azamethiphos at the active site of acetylcholinesterase and hence making the salmon lice resistant towards this OP^29^. Thereafter, we conducted an epidemiological study on a large cohort of salmon lice across the Norwegian coast, leading to the conclusion that the Phe362Tyr mutation was the major genetic factor responsible for azamethiphos resistance in Norway^30^.

Reduced sensitivity or resistance of *L*. *salmonis* towards OPs is not restricted to Norwegian aquaculture, but has also emerged as a major challenge in other salmon producing countries across the North Atlantic Ocean (Canada^31^, Scotland and Shetland Islands^32^). It is however, presently unknown as to whether (i) resistance emerging in salmon lice in other regions of the North Atlantic is also caused by the Phe362Tyr mutation, and (ii) the mechanism(s) by which resistance developed are potentially dispersed over this large geographic region. To address the first question, salmon louse samples, collected over 3 years (2014–2016), from different regions across the North Atlantic, were genotyped for the Phe362Tyr mutation and association of the mutant allele with the survival of the parasite under azamethiphos treatment was determined. The second question was addressed by the genotyping and analysis of temporal samples, collected over 12 years (1998–2009), for a set of nine SNPs located within 5 cM on linkage group 14, where the Phe362Tyr mutation is located.

## Results

### Genetic data

**Figure 1:**
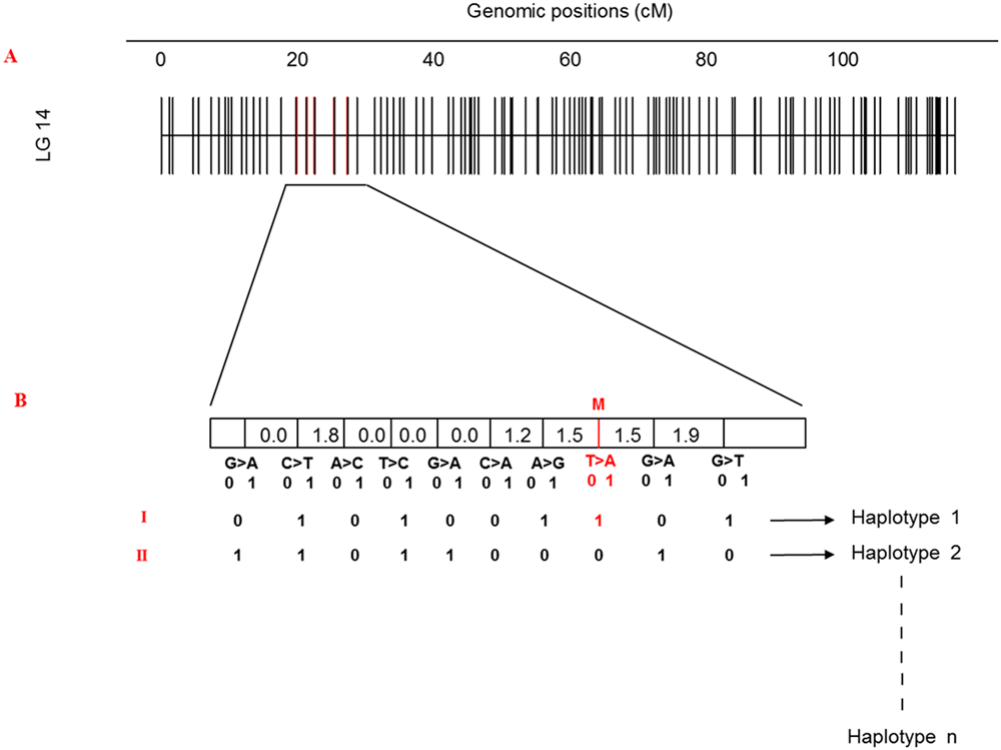
The nine SNPs employed for haplotype reconstruction. (**A**) The location of nine SNPs used for haplotype analysis on LG14 is shown. (**B**) The zoom in view of the SNPs, where *M* denotes Phe362Tyr. The alleles of SNPs were randomly assigned either *0* or *1*. I and II represents the examples where haplotypes were generated based on different combinations of SNPs.

Two sample sets, Sample set I (Supplementary File S1) and Sample set II (Supplementary File S2), were genotyped in the present study. In Sample set I, a total of 1287 *L*. *salmonis*, collected from different regions across the North Atlantic over three years (2014–2016), were screened for Phe362Tyr using the TaqMan assay developed for high throughput screening of this mutation. In Sample set II, a total of 1036 *L*. *salmonis*, from across the North Atlantic, were genotyped for a number of SNPs flanking the Phe362Tyr mutation using an Sequenom MassARRAY Analyser. These samples were taken over a 12-year period, with the earliest sample collected from Norwegian farmed salmon in 1998. Of the 12 SNPs tightly flanking the Phe362Tyr mutation that were originally selected for genotyping (Supplementary File S3), three had to be discarded due to poor amplification or unreliable genotyping. The remaining nine flanking SNPs gave reliable clustering and were utilized for the study. The raw genotype data are available in Supplementary File S4. The exact location of these nine flanking SNPs in relation to Phe362Tyr on chromosome 14^33^ is presented (Fig. 1). Some of the samples in Sample set II had previously been genotyped for the nine flanking SNPs (Supplementary File S2) using the SNP chip developed in Besnier *et al*.^33^. We were able to use these data to cross-validate genotyping accuracy here.

### Frequency of the Phe362Tyr mutant allele across the North Atlantic

**Figure 2:**
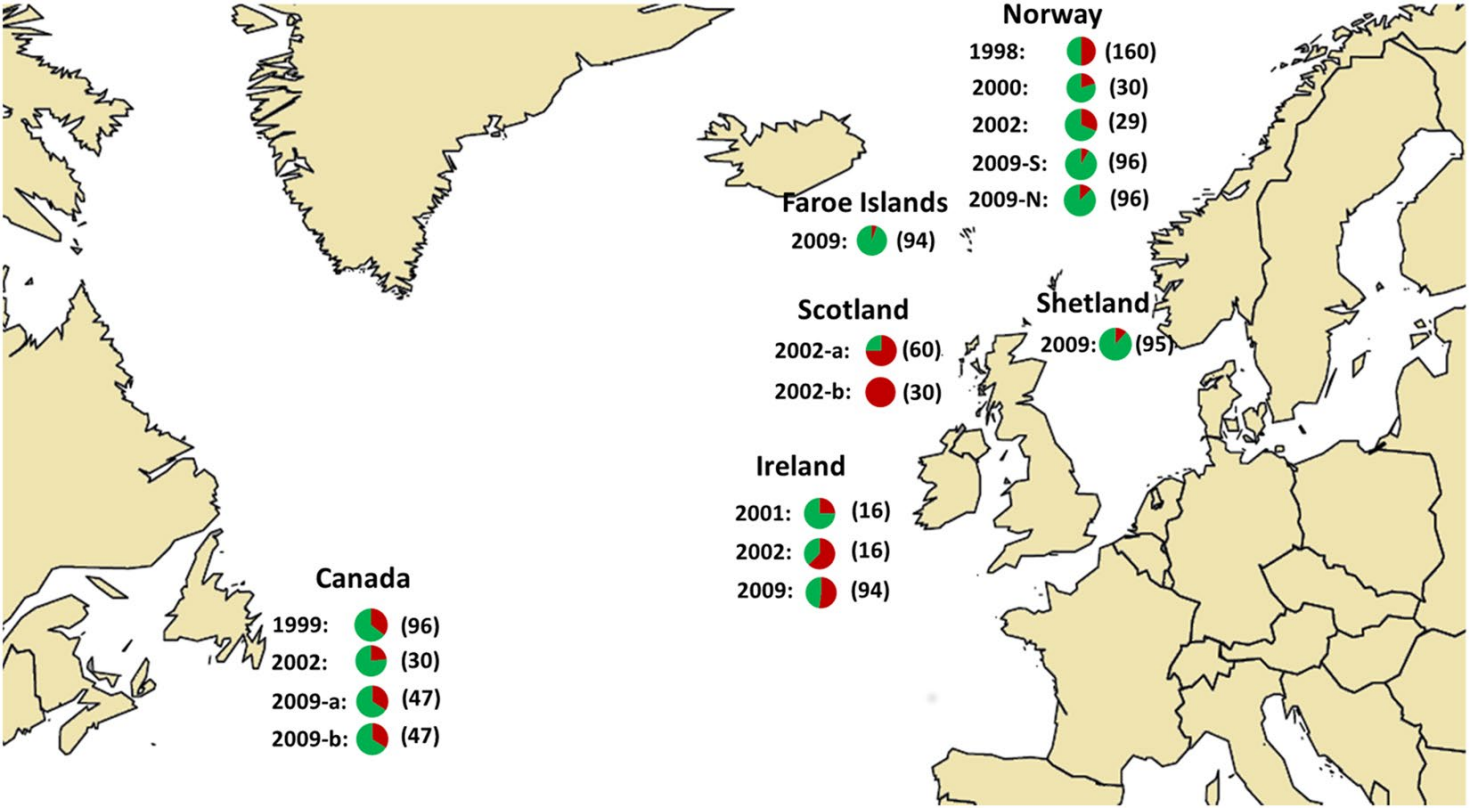
The distribution of the Phe362Tyr mutation in the historical salmon louse samples from the North Atlantic. All samples are collected from farmed Atlantic salmon. The red color of the pie charts represent the frequency (in percentages) of mutant allele observed in these historical samples. Numbers in parenthesis denotes the total number of salmon louse in each respective geographical area. The maps were generated using the software & programming language R^46^.

The mutation giving rise to azamethiphos resistance in *L*. *salmonis*, was present in high frequencies in all of the samples (termed as historical samples hereafter) collected in past years across different salmon farms in the North Atlantic (Fig. 2). This confirms that Phe362Tyr is not only restricted to Norwegian salmonid farms but also can be found across the North Atlantic Ocean. The highest frequency (in percentages) of Phe362Tyr was observed in samples from Scotland in 2002 (75% in Scotland 2002a and 100% in Scotland 2002b, respectively), followed by samples from Ireland 2009 (62%) and Norway 1998 (50%). The lowest frequency was observed in samples from Norway in 2009 (8% in South Norway and 13% in North Norway, respectively).

### Association of Phe362Tyr with azamethiphos resistance across the North Atlantic

The association of this mutation with resistance towards azamethiphos has previously been established in *L*. *salmonis* collected from Norwegian farms^16^. However, nothing has been published so far about its role in the development of resistance in salmon lice collected outside Norway. In order to address this, we genotyped a series of samples from Scotland, Shetland Islands, and Faeroe Islands collected in the period 2014–2016 (Supplementary File S1). These samples were collected from different fish farms, with some before and some after the azamethiphos treatment. We screened these samples for Phe362Tyr and determined the frequency of the mutant allele using the TaqMan probe assay developed for Phe362Tyr screening^30^. The analysis revealed that samples which were collected from farms after the azamethiphos treatment were either homozygous (RR) or heterozygous (RS) for the Phe362Tyr and none of the samples analyzed harbored the wild type (SS) genotype, indicating clearly that all the survivors of azamethiphos treatment carried the Phe362Tyr mutation (Supplementary File S1). In contrast, samples collected from the farms that were not treated with azamethiphos displayed a higher frequency of the SS genotype (Supplementary File S1). These results demonstrate the role of Phe362Tyr mutation in the survival of the salmon louse under azamethiphos treatment, confirming the association of this mutation with OPs resistance areas across the North Atlantic.

### Origin of the Phe362Tyr mutation

**Figure 3:**
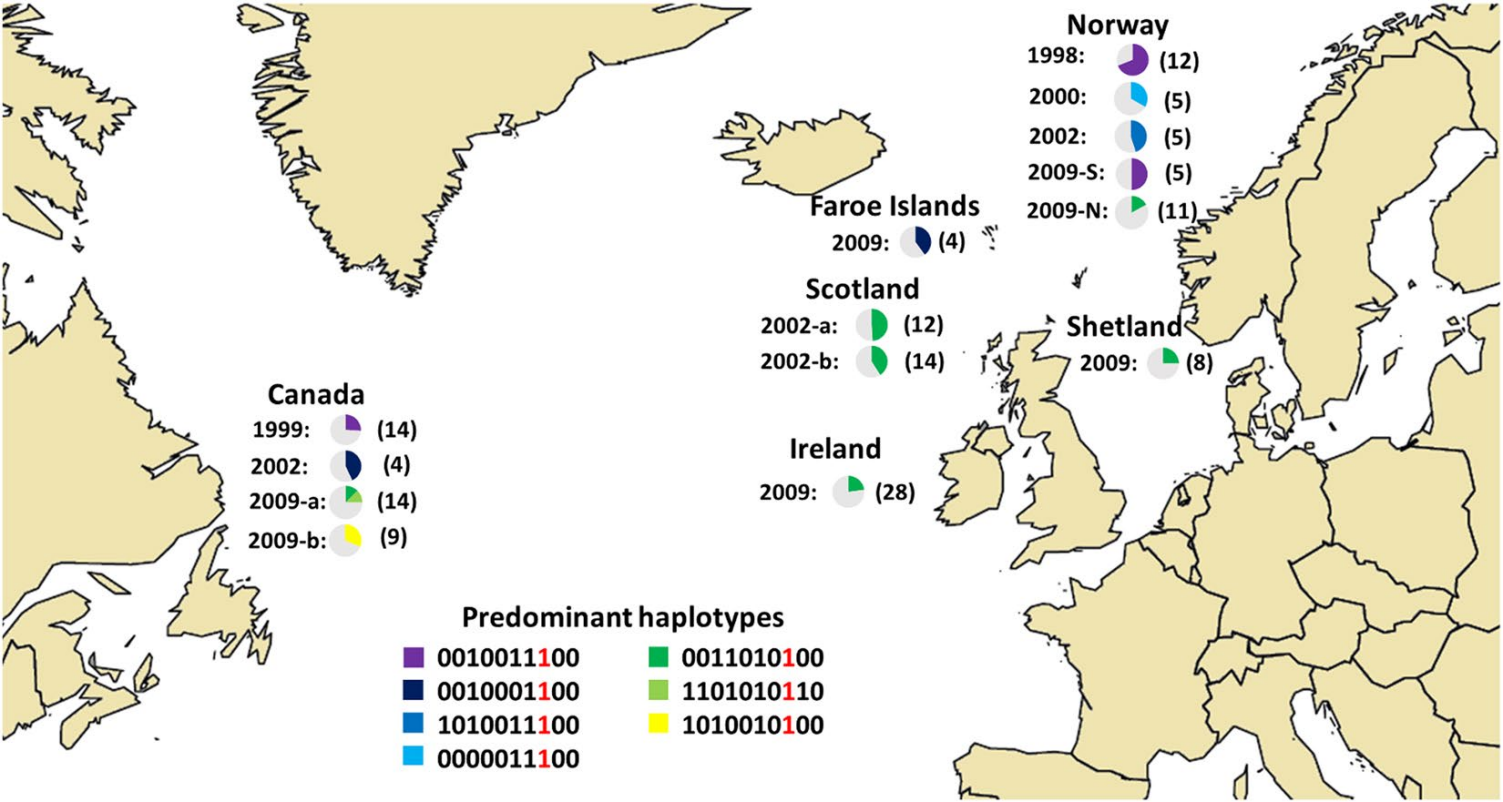
The distribution of the predominant haplotypes carrying the Phe362Tyr mutation. The pie charts represent the frequency of predominant haplotype carrying Phe362Tyr found in different geographical areas. The mutant allele  is marked in red. The numbers in parenthesis denote the number of haplotypes carrying the mutant allele within each sample set. The maps were generated using the software & programming language R^46^.

Using data from the Phe362Tyr mutation and the nine tightly located flanking SNPs, haplotypes were constructed across the 1036 historical lice samples. Lice were classified as resistant or sensitive, based on the presence or absence of the mutant allele. Significantly, the mutant allele was associated with multiple haplotypes throughout the North Atlantic (Fig. 2), although some predominant haplotypes were observed among the samples collected from geographically distant regions (Fig. 3). For example, *0010011100* was the most predominant haplotype in the resistant samples from Norway in 1998, South Norway in 2009, Canada in 1999 and Canada in 2002 at a frequency of 69%, 50%, 26% and 43%, respectively. A nearly identical haplotype *0010001100* was observed at a frequency of 40% in the resistant lice sampled from the Faroe Islands, and another nearly identical haplotype *1010011100* was observed in resistant samples collected in Norway in 2002 at frequency of 45%. Similarly, a common haplotype *0011010100* was shared by the resistant salmon louse samples from Scotland in 2002a, Scotland 2002b and Shetland Islands in 2009 at a frequency of 54%, 41% and 25%, respectively. The predominant haplotypes observed in different resistant samples did not share any common *core region*, strongly suggesting that these haplotypes did not originate from a common founder.

The genotyping data revealed the presence of various haplotypes carrying the mutant allele (Fig. 2). It is highly unlikely that the same mutation would have occurred multiple times in distant geographic regions resulting in these numerous haplotypes. Hence, our data strongly indicate that Phe362Tyr is most likely not a *de novo* mutation and would not have originated due to OPs use.

**Figure 4:**
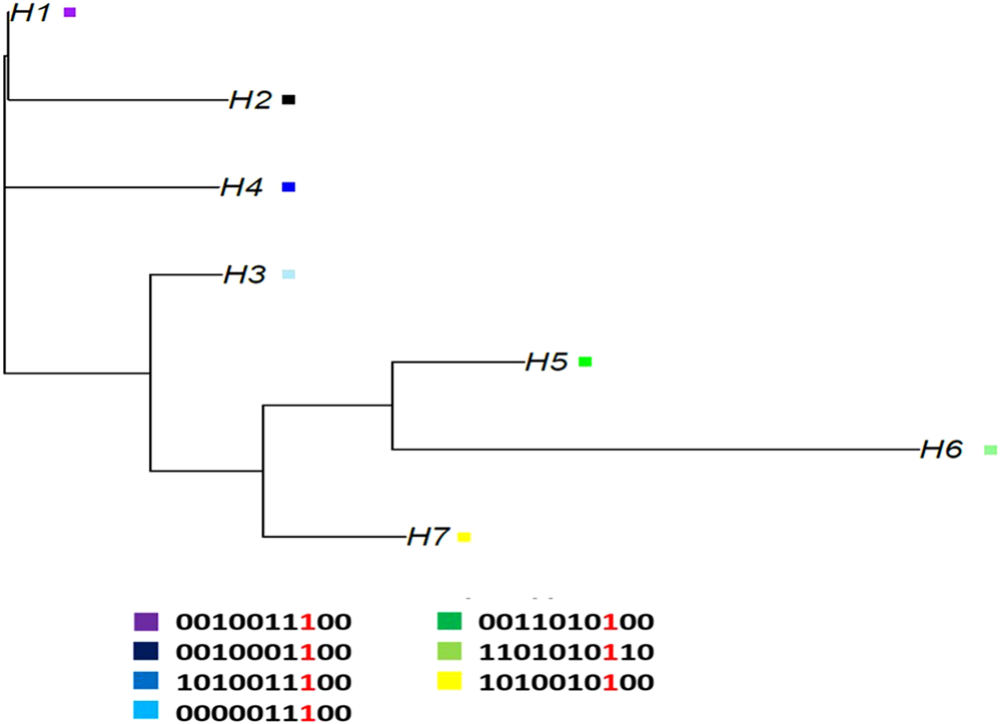
Phylogenetic analysis of resistant haplotypes. The phylogenetic analysis of the resistant haplotypes reveals similarities between genetic and geographical patterns. Haplotype 1, 2 and 3 are clustering together in the same branch of the phylogenetic tree, and are predominant in Norwegian samples. In parallel, haplotype 5, 6 and 7 are also clustering together in the phylogenetic tree, and are predominant in samples from the UK and Canada. Haplotype 6 appears to be both the most distant haplotype on the tree as well as a very singular haplotype that appear only in one of our sample (Canada 2009). Different haplotypes are represented in different colors at the bottom of the phylogenetic tree. The mutant allele is marked in red.

The phylogenetic analysis of the resistant haplotypes reveals similarities between genetic and geographical patterns. As shown in Fig. 4, three haplotypes that were specific for the samples from Norway were clustered together on the phylogenetic tree. Similarly, three haplotypes were specific for samples from the UK (Ireland and Scotland) and were clustered on the same branch of phylogenetic tree. In addition, haplotype 6 appears to be both the most distant haplotype on the tree as well as a very singular haplotype that appears only in one of our samples (Canada 2009).

## Discussion

This is the first study to investigate the presence of the Phe362Tyr, a mutation linked with azamethiphos resistance in *L*. *salmonis* from Norway^30^, in salmon lice sampled across the entire North Atlantic. Together with haplotype re-construction based upon the analysis of SNPs tightly co-located with this gene on linkage group 14, our study provides the following main results: (1) The Phe362Tyr mutation was extensively distributed in *L*. *salmonis* sampled throughout the entire North Atlantic from as early as the late 1990’s and early 2000’s. (2) The Phe362Tyr mutation is strongly associated with survival of salmon lice treated with azamethiphos across the North Atlantic, and (3) Salmon lice displaying the mutant allele at this locus displayed very diverse, high frequency haplotypes (i.e., varies genetically in the tightly located flanking SNPs). Based upon these results, we conclude that Phe362Tyr mutation is the primary cause of azamethiphos resistance in *L*. *salmonis* throughout its entire range in the North Atlantic. We further conclude that this mutation existed in a very low frequency in *L*. *salmonis* throughout the North Atlantic prior to the first use of OPs in commercial aquaculture, and thereafter rapidly increased in frequency in multiple regions in parallel following extensive OPs use.

Studies using extensive sampling regimes^34^, large amounts of genetic data^35^, or a combination of high sample sizes and 1000 s of genetic markers^33^ have all led to the conclusion that *L*. *salmonis* is characterized by a single population throughout the North Atlantic. The observed lack of genetically distinct populations is also supported by the fact that reduced sensitivity to emamectin benzoate; a chemical also used to delouse farmed salmonids, established in a single region, and was thereafter quickly distributed to salmon lice throughout the Atlantic within just a few years^33^. The authors of that work drew such a conclusion as they observed a strong selective sweep on linkage group 5, which displayed few highly conserved haplotypes in salmon lice throughout their Atlantic-wide distribution, and was simultaneously demonstrated to be causatively linked to emamectin benzoate sensitivity in pedigree-based salmon lice^33,36^. The reasoning behind the conclusion was that if resistance had developed in several regions independently (i.e., in parallel), then one would expect diverse as opposed to conserved haplotypes associated with resistance, each haplotype being phylogenetically related to the emergence on the resistance in one region.

**Figure 5:**
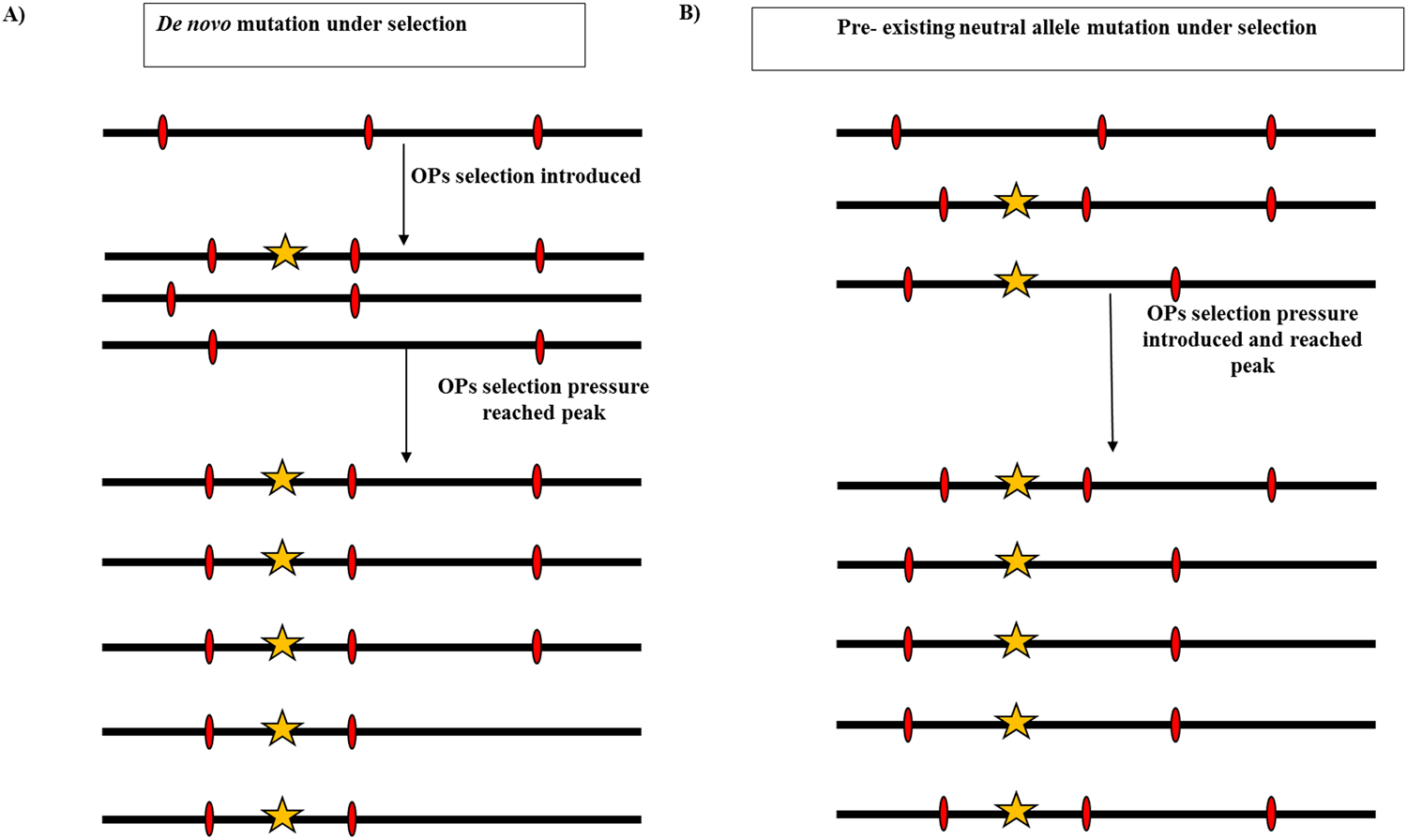
Scenarios for a de novo mutation under selection pressure and a pre-existing neutral allele under selection pressure. The figure presents how a *de novo* mutation arises due to the use of a parasiticide (OPs) on a single haplotype background (**A**) and is selected for in the population resistant for treatment under the selection pressure. The increase in selection pressure results in the increase of the mutation originated and hence the haplotype background carrying the mutant allele. (**B**) Selection pressure on a previously segregated mutation (once as a neutral allele) in the population neutrally and thus existed on multiple haplotype backgrounds. In this way, the neutral allele may carry multiple haplotypes at intermediate frequencies, while moving towards fixation under the selection pressure due to the parasiticide (OPs) use. This figure was adapted from Jensen and coworkers 2014^37^.

The manner by which emamectin benzoate resistance developed (single origin and thereafter rapid dispersal) is in contrast to the way in which the present study concludes OPs resistance developed and dispersed (selection on a low background frequency of an existing mutation, facilitated by dispersal). This begs the question, what evidence supports the conclusion here that the Phe362Tyr mutation existed prior to OPs use? Essentially, there was a lack of pattern in the haplotypes reconstructed around the target mutation. i.e., the SNPs closely linked to the Phe362Tyr mutation on linkage group 14 were very diverse both within and among lice collected from geographically distinct regions. Therefore, the observed haplotypes do not appear to derivate one from another, like coalescent lineages converging toward a single common ancestor, but instead appear like distinct lineages that emerged in parallel when OPs were first used. Over time, recombination erodes the link between neutral flanking SNPs and target genes under selection, and OPs have been used for a much longer period than emamectin benzoate. However, it is unlikely that recombination could have created the large haplotype diversity observed in this study if azamethiphos resistance had developed as a *De novo* mutation during OPs use, as was the case for emamectin benzoate resistance. This is because the period from the introduction of OPs in 1970s, the first reports of OPs resistance in early 1990s, and the early 2000s when the first samples upon which the present study is based were collected, is too short to create the observed diversity. Consequently, it is concluded that the Phe362Tyr mutation, which is responsible for azamethiphos resistance in salmon lice, must have displayed a low or very low frequency in salmon lice distributed throughout the Atlantic prior to OPs use as a *neutral allele*, as opposed to representing a *de novo* mutation during or in response to use of OPs (Fig. 5)^37^. As a neutral allele, without any selection pressure, Phe362Tyr had equal probability of occurring on different haplotype backgrounds. When OPs were first introduced as antiparasitic agents against the salmon louse, parasites carrying the beneficial allele (362Tyr) would have survived chemical treatment on commercial farms. As the selection pressure via use of OPs increased, it led to an increase in frequency of the 362Tyr allele in lice across the North Atlantic in parallel. As a result, the haplotypes carrying the mutant allele became prevalent among the resistant populations. The haplotypes present at initial higher frequencies, before the selection pressure, became the most predominant ones after selection pressure reached its peak (Fig. 5)^37^. This was probably assisted by rapid genetic dispersion as has been previously been demonstrated in this parasite^33^.

Excluding a *de novo* mutation hypothesis during OPs use still leaves us with the possibility that the Phe362Tyr mutation may have independently occurred several times in history prior to OPs use. However, more than 70 different missense mutations are associated with OPs resistance in arthropods^38^. Previously, we only found one mutation (Phe362Tyr) in our samples to be associated with azamethiphos resistance^29^. Therefore, it is very unlikely that the same mutation would have occurred multiple times in different geographical locations.

The increase in frequency of the standing neutral variation, due to its beneficial nature towards changing conditions, which increases chances of host contemporary adaptation in the changing environment, is a common phenomenon and has also been reported earlier in other species. Hartley and co-workers^39^ have provided an unambiguous evidence for this phenomenon in their study on pinned insects (*Lucilia cuprina*). The authors screened the *Lucilia cuprina* samples, collected before the introduction of OPs, for mutations associated with resistance towards OPs. Interestingly 16% of the samples were found to harbor these mutations, clearly showing that the mutations conferring resistance were present at detectable frequencies in the population before the introduction of OPs^39^. In another study, an adaptive transposable element was identified in *Drosophila melanogaster* that expanded and became prevalent due to its beneficial nature towards OPs^40^. However, OPs were not the reason for its origin. These studies support the observations in the present study that resistance associated mutations could pre-date the introduction of parasiticide and could present as *neutral alleles* in the natural population.

## Conclusions

This study provides novel insights into the origin and development of resistance towards OPs in the parasitic salmon louse. We have demonstrated that the Phe362Tyr mutation, the major mechanism for azamethiphos in *L*. *salmonis*, is not only restricted to Norwegian salmon farms. Rather, it is widely spread across the North Atlantic, and is the primary mechanism for azamethiphos resistance in other salmon producing countries such as Scotland, Shetland and the Faeroe Islands. The genotyping analysis of the historical samples from six geographically distant regions across the North Atlantic indicated strongly that Phe362Tyr is not a *de novo* mutation, but was most likely present in salmon lice before the introduction of OPs in commercial aquaculture.

## Methods

### Samples

#### Sample set I

Salmon louse samples were collected from different farms in Scotland (n = 569), Shetland Islands (n = 361) and Faroe Islands (n = 357) between 2014–2016 (Supplementary File S1). The farms faced treatment failures with azamethiphos and consequently sent samples to PatoGen Analyse AS for genetic analysis. The samples were screened for Phe362Tyr, using the TaqMan probe assay^30^ developed by PatoGen Analyse AS, to determine the association of mutant allele with the survival of parasite under azamethiphos treatment and as a result with resistance towards this chemotherapeutant.

#### Sample set II

Salmon louse samples (n = 1036) were collected over a 12-year period (1998–2009) from six geographical regions across the North Atlantic (Fig. 2). Some of these samples originated from previous genetics studies of *L*. *salmonis* (Supplementary File S2), while others were analyzed for the first time in this study (Fig. 2). All the samples were collected from farmed salmon. These samples were used for genetic analysis using nine SNPs flanking the Phe362Tyr to study the origin of the mutant allele.

### RNA extraction

For Sample set I, RNA was extracted from all the salmon louse samples using RNeasy plus Mini kit (Qiagen, CA, USA), followed by genotype analysis using the standard TaqMan assay developed by PatoGen Analyse AS, for the rapid and high throughput screening of Phe362Tyr^30^.

### DNA extraction

For Sample set II, DNA was isolated from all the salmon louse samples in 96-well format using DNeasy kit as per manufacturer’s instructions (Qiagen, Hilden, Germany). A spot-check of DNA quality and quantity was made for some of the samples using an ND-100 Spectrophotometer (Thermo Fisher Scientific, DE, USA). Prior to genotyping, all samples were re-organized onto 384 plate format.

### SNP selection and genotyping

Twelve SNPs flanking the Phe362Tyr mutation site were selected for genotyping. This selection was done on the basis of SNPs being positioned on the recombination map, within a 5 cM (centimorgan) window on each side of the mutation. Unfortunately, genotyping failed for three SNPs. Hence, nine SNPs plus the mutation were used for further haplotype analysis. The exact locations of these nine SNPs in the *L*. *salmonis* recombination map together with flanking sequences are available in Supplementary File S3.

The above chosen SNPs, including the Phe362Tyr mutation, were genotyped in a single multiplex reaction on a Sequenom MassARRAY Analyser instrument at the molecular genetics laboratory at the Institute of Marine Research in Bergen. The MassArray Assay Design software (Agena Bioscience) was used to design the assay using the default values in the software (e.g. amplicon length 80–120 bp, and extension primer length 17–28 bp). Following genotyping, mass signals of a multiplexed extended primer extension spectrum were analyzed using the Typer Analyzer software from Agena Bioscience, and the resulting genotypes exported. All spectrums (clusters) were scored independently by two persons prior to exporting data. For the samples that had previously been analyzed for some of the flanking SNPs in Besnier *et al*.^33^, and analyzed for the patented TaqMan assay for the Phe362Tyr mutation at PatoGen AS, genotypes from the current analysis were compared to their previous analyses for consistency.

### Haplotype analysis

Haplotypes were reconstructed in each sample independently with the Bayesian method implemented in the software Phase2^41,42^, with 1000 burn-in, 1000 iterations, and thinning intervals of 1. Starting from an un-phased set of genotypes (G), the algorithm iteratively estimates the posterior distribution of individual’s haplotypes H given G as Pr(H|G). The procedure consists in repeatedly choosing individuals randomly and estimating its haplotype assuming all the other haplotypes are correctly reconstructed. Repeating this process enough time results in an approximate sample from Pr(H|G)^41^.

The list of resistant haplotypes were imported in R via the package APE^42,43^ using the *read*.*dna* function. A distance matrix between haplotypes was estimated by the *dist*.*dna* function using the Felsenstein (1981)^44^ model, and a phylogenetic tree was obtained from the *bionj* function^45^.

### Ethics Statement

The study involved only salmon louse and no fish were being used. All the parasites were collected from fish farms with farmer’s consent. After farmer’s consent, no further permissions were required for sample collection. The study did not involve endangered or protected species.

## Electronic supplementary material

Supplementary file
