# Supplementary material for: The mechanism (Phe362Tyr mutation) behind resistance in Lepeophtheirus salmonis pre-dates organophosphate use in salmon farming
Source: Sci Rep. 2017 Sep 27;7:12349. doi: 10.1038/s41598-017-12384-6 (PMC5617835; doi:10.1038/s41598-017-12384-6)
Supplement: Supplementary file 1 — Supplementary file [file 41598_2017_12384_MOESM1_ESM.pdf]

The mechanism (Phe362Tyr mutation) behind resistance in *Lepeophtheirus salmonis* pre-dates the use of organophosphate in salmon farming

Kiranpreet Kaur, Francois Besnier, Kevin A Glover, Frank Nilsen, Vidar Teis Aspehaug, Helene Børretzen Fjørtoft, Tor Einar Horsberg

Country	Site code	Date of sample collection	Phe362Tyr			Status of treatment while collection
			SS	RS	RR	
Scotland	1	12.11.2015	24	79	47	Untreated
Scotland	1	20.01.2016	0	45	45	Treated with Azamethiphos
Scotland	5	12.11.2015	24	79	47	Untreated
Scotland	5	20.01.2016	0	45	45	Treated with Azamethiphos
Scotland	3	21.05.2015	0	22	38	Treated with Azamethiphos
Scotland	3	08.07.2015	0	3	26	Treated with Azamethiphos
Shetland Islands	2	04.09.2014	13	25	21	Untreated
Shetland Islands	3	25.06.2014	36	62	24	Untreated
Shetland Islands	4	20.09.2014	0	15	165	Treated with Azamethiphos
Faroe Islands	6	14.04.2016	39	39	12	Untreated
Faroe Islands	7	14.04.2016	0	42	45	Treated with Azamethiphos
Faroe Islands	9	09.10.2015	0	54	33	Treated neighbouring site
Faroe Islands	10	12.10.2015	69	18	3	Untreated

Supplementary table S1: Details of sample set I

Total N= 1287

Supplementary table S2: Details of sample set II

Country	Year	total samples	Present study/Previous study [33]
Norway	1998	160	present study
Norway	2000	30	[33]
Norway	2002	29	[33]
North Norway	2009	96	[33]
South Norway	2009	96	[33]
Canada	1999	96	present study
Canada	2002	30	[33]
Canada (a)	2009	47	[33]
Canada (b)	2009	47	[33]
Scotland (a)	2002	60	present study
Scotland (b)	2002	30	[33]
Shetland	2009	95	[33]
Ireland	2001	16	present study
Ireland	2002	16	present study
Ireland	2009	94	[33]
Faroe Islands	2009	94	[33]
		N= 1036	

Supplementary table S3: SNPs selected for genotyping

LG	name	flanking	SNP	flanking	map position
14	LS_Gdist_c19637.22906	AATCTTGACCCCCTCATAAATAGGAACTCCCATGAGGATGAAATCTTTGACGACTACATTACGAAACTGACTTTGACGCAAAAAGGAAGGTCTTTCAAA	[T/C]	AATGTATCTTTCATTGTATTACGTTAGCTTGATTGGAAGTAATTTAGTTTTCATGCATGNNNNNNNNNNNNNNNNN	19,8
14	LS_Gdist_c9990.2279	CTATTATGGCAAAAAAATATCTTCTCACATATTATTTCAGGTTCAAAATAGGTTTCCTTTCACTTGAAATCAATTTTAATGGAGTATTACGTTCATTTT	[A/G]	CAAACATCGTGTATCAATTCCTTCAAGAGTGAGGTAGCCTTCGTCTCCCTGACAGGTTTAGTGAGTTTTAACCGGAGTCGGAGTCACAAAATTCCTAAAA	19,8
14	LS_Gdist_c7174.6841	TTTAATATCCGAAAAGTTCCTCAATCTTATTATGATCTGTAAAAGGTTTGGGTACGGGAAGCCGGTTCGGATCTTGGATCATTTTGGATACGGGGTAAT	[A/G]	CATTTATGACGTCTTAAAAAAGTACAATTGTTAAATCTGTGTGTATTTGTCCAAAACTTGATATCCCCCCCCCCTCCGATTGGACTCATTAACTCACACC	21,3
14	LS_Gdist_c7508.2175	TAGCTGGAGAAAATTATATATGAGCCTCAGAATCTTCCTCTAGATTTCCTCCATTTGATTTTAACTACGACTATTTTGAGGGAGGACTTTTAATAATGA	[T/C]	GTTATTCATCTTAATTATTTATTATATGAAAATATCGCTGAAATGAACAACGCTGAAAAAAAGCTCGCCCAAATAAATAAATGCTCAAATGAATGTTGGT	21,3
14	LS_Gdist_c14196.14756	TAATCAACCCCTGGGTCTGGGGTTTATTTTGAAGTGTTTGGAGGGAGCCTAAATGCTTAGTGAAGTAACGGATGGAATTTTAACCTGCATGGTGTCCCT	[A/G]	]TCGTAGTCCTAAACAATCAATGGACCGGGGGTTCATTTTGAAGCATTTGGAGGGGTTCAAAGGCTTTGAGAAGTGGTTGCAGTAGTTTATAACCTGAATA	21,3
14	LS_Gdist_c7446.141775	CAATTAAATTTTCCATCAATTGAAAACCTTTATAAACTAGCATAATTTAGAAGGAA	[T/C]	ACTGAGCATTCATTTTTTACCCATATATATCCAACTTTAGATTGTTAACATAATTTAGAGTAGAATTATGTTTAGACTTATACA	21,3
14	LS_Diag_c7446.181381	AACAAAAATAGAACCAAGAGGAAACTTCCAATTATGAGCTGTAAATAAAAAAAAACCCAGCAAAATGGAACAAAGAGCAGCATTTAAGGCACAAAAATTA	[T/C]	AAGACAAGATTGAAAGGCTACAGAAAGGATGTTCGTACACATTTGAGGGTACTTCATCCAAATATTCTGCATTCAAAGATGATTTTTTTTTGAGTTATTG	22,5
14	Target	ATGATGAGCTGTCCTTACGATGAAAAAAATACAAAGGCCATGATTGAATGCCTGCGGCAAAAGGATGCAACAGAGATGGTTAATCAGGAGTGGATTGGTATCATTTCTGGGATTGCAGAGTTCCCTTTTGTTCCTATTGTGGATGTCTTGAAGTTTCTTGGATGAGAGCCCTGGAAAATCTCTTACAACAAAGA<br>ACTATAAAAAAACCAACATTTTAATTGGAGCGAATAAGGAAGAAGGGAATTATT	[A/T]	CATCATGTACTATCTTACAGATCTCTTTAAAAATACGGAGAGCGTCTATGTTGATCGAACAGATTTCATTCGAAGTGTTTCAGAATTGAACCACTATGTAAAAAAAAATGGGAAGAGAGGCAATAACTTTTGAGTACACAGATTGGCTCAATCCAAACGATCCCATAAAAAATAGAGAAGCAATTGATCGCATGGTCGGTGACTATCAATTCATTTGCCCAACTGCTGACTTTGCT	
14	LS_Diag_c64652.27830	TCAATTTGTCCAAAAAAGATGCTTGACTTTGTTTTTGACCTTTTTGAAAGTATCTCTTGATCTTGAATTGAGTTACATGAAGCATTTTTATTGAACCGA	[T/C]	GTAAGTACACC	25,4
14	LS_Diag_c14769.22573	TGATAAAAAAATGGCAAAATATTGAGTTTTAGTAAGTTATTTATAAGATGAATCTAGGGATTGAATAAATAATACGTAACAAAATACTTTAAATAAATAG	[T/C]	GGAGCAATTGGGGAGGTCAAAAGAAGTCGGATCGTCATTTTTTAGCTATGTCATTAATTTCAATTAGTAAAAATGTTTGTATGATTGTTAAAAAAGTTAA	27,3
14	LS_Gdist_c14769.23700	ACAGAGTAATTATTTAATAGTTGTTGTCTGAATATTTAGGACGTAATATTTATTTATGACGCAAAATAGGTGTTCGATTTTGTACAAAATAGTAACCAA	[T/C]	GCAATTGTTCCAGTTTATTNNNNNNNNNNNNNNNNNNNNNNNNNNNNNNNNNNNNNNNNNNNNNNNNNNNNNNNNNNNNGGTACACGTAAATATTACTGTTAAG	27,3
14	LS_Gdist_c8326.48240	ATGTTGTACTTCTTTAAAAACTATAATACAATACATAGTTCCTACTCAATGATTCCGAAATCCTTTCCTCTCCTCACGTTGTTCCAACTGTGTGAGAAA	[T/C]	GTTCATTTTATCAAAGACACAATTACGTACGTTTAACATACAATCAAATCACAAATAAAACATTATTTTTAAGTGGGGGAGGGGGGGGGGAAGATGAAGT	27,3
14	LS_Gdist_c8039.18856	ATTAGAGCTTGAATAAATTCCAAACAAATTAACGTATTATGAAACATACATTGTTTGAAAGAGTAGTACTTTTTTCCTACCTCTAATTATCTGTAAACT	[A/G]	TTTATGAGTTTATGAGTGCGTTGATCGACTGTATCCTCAAACCTCGATCTGCAAGTGCACAAAAAAACACAACGTTATTGAAGGGACTATCAAGGATACA	28,7

Supplementary table S4: Raw genotype data

Country_ year	SAMPLE NAME	SNP_ Gdist_ c1963722906	SNP_ Gdist_ c99902279	SNP_ Gdist_ c71746841	SNP_ Gdist_ c75082175	SNP_ Gdist_ c1419614756	SNP_ Gdist_ c7446141775	SNP_ Diag_ c7446181381	Target (Phe362Tyr)	SNP_ Gdist_ c832648240	SNP_ Gdist_ c803918856
South Norway-2009	1603_813_1	C	G	GA	CT	G	TC	CT	T	C	
South Norway-2009	1603_813_10	CT	G	GA	CT	A	T	T	T	CT	
South Norway-2009	1603_813_11	C	GA	A	CT	A	T	CT	T	C	G
South Norway-2009	1603_813_12	C	G	A	C	G	TC	CT	T	C	
South Norway-2009	1603_813_13	CT	G	GA	T	G	TC	C	T	CT	G
South Norway-2009	1603_813_14	C	G	GA	CT	G	TC	C	T	C	G
South Norway-2009	1603_813_15	C	G	G	CT	GA	T	CT	T	C	G
South Norway-2009	1603_813_16	C	G	GA	C	G	C	CT	T	C	G
South Norway-2009	1603_813_17	C	GA	G	CT	G	T	CT	T	C	G
South Norway-2009	1603_813_18	C	G	A	CT	G	C	C	T	CT	
South Norway-2009	1603_813_19	C	GA	G	CT	G	T	CT	T	CT	
South Norway-2009	1603_813_2		GA	GA	CT	G	TC	CT	T	C	G
South Norway-2009	1603_813_20	CT	GA	GA	CT	G	C	CT	T	CT	G
South Norway-2009	1603_813_21	C	G	GA	CT	A	C	CT	T	C	
South Norway-2009	1603_813_22	CT	G	GA	CT	G	T	C	T	T	
South Norway-2009	1603_813_23	C	A	GA	C	G	TC	C	T	C	G
South Norway-2009	1603_813_24	CT	A		CT	G	TC	CT	T	C	
South Norway-2009	1603_813_25	C	A	G	T	G	C	CT	T	C	G
South Norway-2009	1603_813_26	C	GA	G	CT	G	T	CT	T	CT	G
South Norway-2009	1603_813_27	C	GA	GA	T	G	C	CT	T	C	G
South Norway-2009	1603_813_28	C	GA	GA	CT	G	T	CT	T	C	
South Norway-2009	1603_813_29	CT	GA	GA	CT	G	T	T	T	C	
South Norway-2009	1603_813_3	T	GA	G	C	G	TC	C	T	T	G
South Norway-2009	1603_813_30	CT	A	G		G	C	CT	T	CT	G
South Norway-2009	1603_813_31	C	GA	G	T	G	T	T	T	C	

South Norway-2009	1603_813_32	C	G	GA	C	G	TC	CT	T	T	G
South Norway-2009	1603_813_33	CT	G	GA	CT	G	T	CT	T	C	
South Norway-2009	1603_813_34	CT	GA	GA	C	G	TC	C	T	CT	G
South Norway-2009	1603_813_35	C	GA	GA	CT	G	TC	T	T	C	
South Norway-2009	1603_813_36	CT	G	GA	C	A	C	CT	TA	C	G
South Norway-2009	1603_813_37	T	G	GA	T	G	TC	CT	T	CT	G
South Norway-2009	1603_813_38	C	G	GA		G	C	T	TA	C	G
South Norway-2009	1603_813_39	CT	G	G	CT	G	T	CT	T	CT	G
South Norway-2009	1603_813_4	C	G	GA	CT	G	TC	CT	T	C	G
South Norway-2009	1603_813_40	C	A	G	CT	A	TC	C	T	CT	
South Norway-2009	1603_813_41	C	G	G	T	G	T	CT	T	C	G
South Norway-2009	1603_813_42	C	GA	G	T	G	TC	CT	T	CT	G
South Norway-2009	1603_813_43	CT	GA	G	T	G	TC	T	T	C	G
South Norway-2009	1603_813_44	C	G	GA	T	A	T	T	T	CT	
South Norway-2009	1603_813_45	T	G	GA	C	G	TC	CT	T	C	G
South Norway-2009	1603_813_46	C	G	GA	CT	G	TC	CT	T	C	
South Norway-2009	1603_813_5	CT	G	G	T	G	T	T	T	C	G
South Norway-2009	1603_813_57	CT	GA	GA	C	G	TC	C	T	C	
South Norway-2009	1603_813_58	CT	A	G	CT	G	TC	CT	T	CT	
South Norway-2009	1603_813_6	C	GA	GA	CT	G	T	CT	T	C	
South Norway-2009	1603_813_7	CT	G	GA	CT	G	T	CT	T	CT	G
South Norway-2009	1603_813_8	C	A	GA	CT	G	T	CT	T	C	
South Norway-2009	1603_813_9	C	G	GA	CT	G	TC	C	T	C	
North Norway-2009	1746_837_1	T	GA	GA	C	A	TC	C	T	CT	
North Norway-2009	1746_837_10	C	GA	G	CT	A	TC	CT	T	C	
North Norway-2009	1746_837_11	CT	G	A	C	G	T	C	T	CT	
North Norway-2009	1746_837_12	T	G	GA	T	G	TC	CT	T	C	
North Norway-2009	1746_837_13	C	G	GA		A	TC	T	T	C	
North Norway-2009	1746_837_14	C	G	GA	C	G	TC	CT	TA	C	
North Norway-2009	1746_837_15	C	GA	A	CT	G	C	C	T	C	G
North Norway-2009	1746_837_16	C	G	G	T	A	TC	CT	T	CT	
North Norway-2009	1746_837_17	C	G	GA	CT	A	TC	C	T	CT	
North Norway-2009	1746_837_18	C	GA	A	CT	G	TC	C	T	CT	
North Norway-2009	1746_837_19	C	G	GA	CT	G	T	C	T	C	
North Norway-2009	1746_837_2	CT	G	G	CT	G	C	C	T	C	G
North Norway-2009	1746_837_20	C	G	G	CT	G	T	C	T	T	G
North Norway-2009	1746_837_21	C	A	G	T	G	C	C	T	CT	G
North Norway-2009	1746_837_22	C	G	A		G	C	CT	TA	C	A
North Norway-2009	1746_837_23	C	G	A	C	G	T	C	T	C	

North Norway-2009	1746_837_24	CT	G	GA	C	G	TC	CT	TA	CT	G
North Norway-2009	1746_837_25	C	G	G	CT	A	TC	CT	T	C	G
North Norway-2009	1746_837_26	C	GA	G	C	G	TC	C	T	CT	G
North Norway-2009	1746_837_27	C	G	GA	CT	A	TC	C	T	T	
North Norway-2009	1746_837_28	C	A	GA	CT	A	C	C	TA	CT	
North Norway-2009	1746_837_29	T	G	G	CT	G	T	C	T	C	
North Norway-2009	1746_837_3	C	G	GA	C	G	T	C	T	CT	G
North Norway-2009	1746_837_30			A					T		
North Norway-2009	1746_837_31							C			
North Norway-2009	1746_837_32	CT	GA	GA	C	G	TC	C	T	C	A
North Norway-2009	1746_837_33	C	GA	G	CT	G	TC	CT	T	C	G
North Norway-2009	1746_837_34	C	G	GA	CT	G	T	CT	T	CT	
North Norway-2009	1746_837_35	CT	G	A	CT	A	TC	CT	T	CT	G
North Norway-2009	1746_837_36	CT	GA	GA	CT	G	TC	CT	T	CT	
North Norway-2009	1746_837_37	C	GA	GA	C	G	C	C	T	C	A
North Norway-2009	1746_837_38	C	G	GA	C	G	T	C	T	T	
North Norway-2009	1746_837_39	C	GA	A	CT	G	TC	C	TA	C	
North Norway-2009	1746_837_4	C	G	GA	C	GA	TC	CT	TA	C	
North Norway-2009	1746_837_40	C	G	A	C	G	TC	CT	T	C	
North Norway-2009	1746_837_41	C	G	GA	T	G	C	CT	T	CT	
North Norway-2009	1746_837_42	C	G	G	C	A	T	C	T	C	G
North Norway-2009	1746_837_43	CT	G	G	C	A	C	C	T	C	G
North Norway-2009	1746_837_44	C	GA	GA	C	G	TC	C	T	C	A
North Norway-2009	1746_837_45	C	GA	G	C	G	C	CT	T	CT	G
North Norway-2009	1746_837_46	CT	GA	GA	C		T	CT	T	CT	
North Norway-2009	1746_837_47	C	G	G	CT	G	T	CT	T	C	G
North Norway-2009	1746_837_5	CT	GA	GA	C	A	TC	C	T	C	
North Norway-2009	1746_837_50	CT	GA	G	CT	G	T	C	T	CT	
North Norway-2009	1746_837_6	CT	GA	GA	CT	G	C	C	TA	C	
North Norway-2009	1746_837_7	CT	GA	G	CT	A	TC	C	T	T	G
North Norway-2009	1746_837_8	CT	G	G	C	G	TC	C	T	C	G
North Norway-2009	1746_837_9	C	G	A	CT	G	TC	C	TA	C	G
South Norway-2009	1765_854_1	C	GA	A	CT	G	TC	CT	TA	T	G
South Norway-2009	1765_854_10	CT	G		CT	G	TC	C	T	CT	G
South Norway-2009	1765_854_11	CT	GA	GA	T	A	TC	CT	TA	CT	G
South Norway-2009	1765_854_12	CT	G	GA		G	TC	T	TA	C	
South Norway-2009	1765_854_13	CT	A	GA	CT	G	C	C	T	C	
South Norway-2009	1765_854_14	T	G	GA	C	G	TC	T	T	C	G
South Norway-2009	1765_854_15	C	G	GA	CT	G	C	CT	T	C	G

South Norway-2009	1765_854_16	C	GA	G	T	G	T	CT	T	C	G
South Norway-2009	1765_854_17	C	G	A	C	G	C	CT	TA	CT	
South Norway-2009	1765_854_18	C	A	G	T	G	T	T	T	CT	
South Norway-2009	1765_854_19	CT	G		CT	A	T	C	T	CT	G
South Norway-2009	1765_854_2	C	G	G	C	G	C	C	T	CT	
South Norway-2009	1765_854_20	C	GA	A	CT	A	C	C	T	CT	A
South Norway-2009	1765_854_21	C	G	G	CT	G	TC	C	T	C	
South Norway-2009	1765_854_22	C	G	G	C	A	TC	C	T	C	G
South Norway-2009	1765_854_23	C	G	GA	C	G	T	C	T	T	G
South Norway-2009	1765_854_24	C	G	G	C	G	TC	CT	T	CT	G
South Norway-2009	1765_854_25	CT	G	GA	C	A	TC	C	T	CT	G
South Norway-2009	1765_854_26	C	G	G	T	G	T	CT	T	C	G
South Norway-2009	1765_854_27	C	G	GA	CT	G	C	C	T	CT	
South Norway-2009	1765_854_28	CT	GA	G	C	G	TC	CT	T	C	
South Norway-2009	1765_854_29	C	G	A	CT	G	TC	C	T	C	G
South Norway-2009	1765_854_3	CT	G	GA	C	G	C	C	T	C	
South Norway-2009	1765_854_30	CT	G	GA	CT	G	T	CT	T	CT	
South Norway-2009	1765_854_31	C	G	GA	CT	G	TC	C	T	C	G
South Norway2009	1765_854_32	C	GA	GA	CT	G	C	CT	T	CT	
South Norway-2009	1765_854_33	C	A	G	CT	G	T	CT	T	C	G
South Norway-2009	1765_854_34	C	G	GA	C	A	C	CT	T	C	G
South Norway-2009	1765_854_35	C	GA	GA	C	G	C	C	T	C	G
South Norway-2009	1765_854_36	C	GA	G	CT	G	T	CT	T	C	G
South Norway-2009	1765_854_37	C	G	A	CT	G	T	CT	TA	T	A
South Norway-2009	1765_854_38	C	G	GA	CT	A	C	C	T	T	
South Norway-2009	1765_854_39	T	GA	GA	CT	A	T	C	T	CT	
South Norway-2009	1765_854_4	T	GA	G	C	G	TC	C	T	C	G
South Norway-2009	1765_854_40	CT	GA	A	CT	G	TC	C	T	CT	
South Norway-2009	1765_854_41	C	G	GA	T	A	TC	CT	T	C	
South Norway-2009	1765_854_42	C	G	A	C	G	T	C	T	CT	
South Norway-2009	1765_854_43	CT	G	G	CT	G	TC	CT	T	T	A
South Norway-2009	1765_854_44	C	G	G	CT	G	TC	CT	T	C	
South Norway-2009	1765_854_45	CT	G	A	C	G	C	CT	T	C	G
South Norway-2009	1765_854_46	C	A	G		G	TC	T	T	C	G
South Norway-2009	1765_854_47	C	A	GA	C	A	C	CT	T	CT	
South Norway-2009	1765_854_5	CT	G	GA	C	G	TC	CT	TA	C	G
South Norway-2009	1765_854_59	C	GA	GA	C	G	C	CT	T	T	
South Norway-2009	1765_854_6	CT	GA	GA	C	G	C	C	T	CT	G
South Norway-2009	1765_854_7	C	GA	G	C	G	TC	C	T	CT	G

South Norway-2009	1765_854_8	CT	GA	GA	C	G	TC	C	T	T	G
South Norway-2009	1765_854_9	C	G	GA	CT	A	T	C	T	C	
North Norway-2009	1766_849_1	T	G	GA	CT	G	TC	C	T	C	G
North Norway-2009	1766_849_10	C	GA	A	CT	G	C	C	T	CT	G
North Norway-2009	1766_849_11	C	G	G	T	G	T	C	T	CT	
North Norway-2009	1766_849_12	C	GA	A	T	G	TC	C	T	C	
North Norway-2009	1766_849_13	C	G	G	C	G	T	C	T	C	
North Norway-2009	1766_849_14	C	A	G	C	G	TC	C	T	C	
North Norway-2009	1766_849_15	CT	G	GA	CT	G	TC	C	T	CT	
North Norway-2009	1766_849_16	T	GA	G	T	G	TC	CT	T	CT	G
North Norway-2009	1766_849_17	CT	G	A	CT	G	TC	C	T	C	G
North Norway-2009	1766_849_18	CT	G	GA	CT	G	TC	T	T	C	
North Norway-2009	1766_849_19	C	G	GA	C	A	T	C	T	CT	G
North Norway-2009	1766_849_2	C	GA	GA	T	G	TC	CT	T	CT	
North Norway-2009	1766_849_20	C	GA	G	CT	G	T	T	T	CT	
North Norway-2009	1766_849_21	CT	GA	GA	CT	G	TC	C	T	C	A
North Norway-2009	1766_849_22	C	G	GA	C	G	C	CT	T	C	A
North Norway-2009	1766_849_23	C	G	A	CT	G	TC	C	T	C	
North Norway-2009	1766_849_24	CT	G	G	CT	G	TC	C	T	CT	G
North Norway-2009	1766_849_25	C	GA	GA	T	G	TC	C	T	CT	
North Norway-2009	1766_849_26	C	G	GA	CT	A	C	C	T	CT	G
North Norway-2009	1766_849_27	C	GA	GA	C	G	TC	CT	TA	C	G
North Norway-2009	1766_849_28	C	G	GA	CT	G	T	C	T	T	G
North Norway-2009	1766_849_29	CT	G	GA	C	G	TC	C	T	C	A
North Norway-2009	1766_849_3	C	G	GA	CT	A	TC	C	T	C	
North Norway-2009	1766_849_30	C	GA	A	CT	A	TC	C	TA	CT	A
North Norway-2009	1766_849_31	CT	GA	G	CT	G	TC	CT	T	CT	G
North Norway-2009	1766_849_32	CT	GA	G	CT	A	TC	C	T		G
North Norway-2009	1766_849_33	CT	G	GA	T	G	C	C	T	C	
North Norway-2009	1766_849_34	C	GA	GA	C	G	TC	C	T	T	G
North Norway-2009	1766_849_35	C	GA	GA	C	A	C	C	T	C	G
North Norway-2009	1766_849_36	C	G	GA	CT	G	T	CT	T	C	G
North Norway-2009	1766_849_37	C	G	GA		G	TC	CT	TA	T	G
North Norway-2009	1766_849_38	C	G	GA	CT	G	TC	C	TA	CT	
North Norway-2009	1766_849_39	C	GA	GA	CT	G	T	C	T	C	
North Norway-2009	1766_849_4	C	G	GA	C	A	TC	C	T	C	
North Norway-2009	1766_849_40	C	GA	GA	CT	A	TC	C	T	CT	G
North Norway-2009	1766_849_41	C	G	A	CT	A	C	CT	T	CT	G
North Norway-2009	1766_849_42	C	A	GA	CT	G	TC	CT	T	C	G

North Norway-2009	1766_849_43	CT	A	G	T	G	T	CT	T	CT	G
North Norway-2009	1766_849_44	C	G	G	C	G	TC	CT	T	CT	
North Norway-2009	1766_849_45	C	GA	G	C	G	T	C	T	C	
North Norway-2009	1766_849_46	CT	G	A	CT	G	TC	C	T	CT	
North Norway-2009	1766_849_47	C	G	A	CT	A	TC	C	T	C	
North Norway-2009	1766_849_5	C	GA	G	C	A	C	C	T	CT	G
North Norway-2009	1766_849_50	C	GA	G	C	G	T	C	T	CT	G
North Norway-2009	1766_849_6	C	G	A		G	C	CT	T	C	
North Norway-2009	1766_849_7	C	GA	GA	C	G	TC	CT	T	CT	G
North Norway-2009	1766_849_8	C	GA	GA	CT	G	TC	CT	T	C	G
North Norway-2009	1766_849_9	C	G	A	CT	A	C	CT	T	CT	G
Shetland-2009	1771_856_51										
Ireland-2009	1772_852_1	CT	G	GA	C	G	TC	C	T	CT	G
Ireland--2009	1772_852_10	C	G	G		G	TC	T	T	CT	G
Ireland--2009	1772_852_11	CT	G	GA	C	G	TC	CT	T	C	G
Ireland--2009	1772_852_12	C	GA	GA	CT	G	TC	C	T	C	
Ireland--2009	1772_852_13	T	G	A	CT	A	C	C	T	CT	G
Ireland--2009	1772_852_14	T	G	A	CT	G	C	C	T	C	G
Ireland--2009	1772_852_15	C	GA	A	CT	G	TC	CT	TA	C	G
Ireland--2009	1772_852_16	C	G	GA	T	G	C	C	T	C	G
Ireland--2009	1772_852_17	CT	G	G	C	A	TC	C	T	C	
Ireland--2009	1772_852_18	C	GA	GA	C	G	TC	C	T	CT	
Ireland--2009	1772_852_19	C	G	A	C	A	TC	CT	T	CT	G
Ireland--2009	1772_852_2	C	G	G	CT	G	TC	CT	T	CT	
Ireland--2009	1772_852_20	C	G	GA	C	A	T	CT	T	C	G
Ireland--2009	1772_852_21	C	G	G	CT	G	TC	CT	T	CT	G
Ireland-2009	1772_852_22	C	GA	A	T	G	C	C	T	CT	
Ireland--2009	1772_852_23	C	G	A	CT	G	C	C	T	C	G
Ireland--2009	1772_852_24	CT	GA	A	CT	G	C	C	T	CT	G
Ireland--2009	1772_852_25	C	G	A		A	C	CT	T	C	
Ireland--2009	1772_852_26	C	G	GA	C	G	TC	C	T	CT	G
Ireland--2009	1772_852_27	CT	G	A	T	G	C	C	T	CT	G
Ireland--2009	1772_852_28	CT	G	GA	T	G	TC	T	TA	C	G
Ireland--2009	1772_852_29	C	GA	A	T	G	C	C	T	C	
Ireland--2009	1772_852_3	C	G	A	CT	G	C	C	T	CT	
Ireland--2009	1772_852_30	C	G	GA	CT	G	T	CT	T	C	A
Ireland--2009	1772_852_31	C	GA	GA	T	G	C	C	T	C	
Ireland-2009	1772_852_32	CT	G	A	C	GA	TC	C	T	CT	G
Ireland--2009	1772_852_33	CT	GA	A	C	A	TC	C	T	T	G

Ireland--2009	1772_852_34	C	G	A	T	G	C	CT	T	CT	G
Ireland--2009	1772_852_35	T	G	G	C	A	TC	T	T	CT	G
Ireland--2009	1772_852_36	CT	G	A	T	G	TC	C	T	CT	G
Ireland--2009	1772_852_37	CT	G	A	CT	G	T	C	T	CT	G
Ireland--2009	1772_852_38	C	G	GA	CT	G	TC	C	T	C	
Ireland--2009	1772_852_39	CT	GA	GA	T	G	TC	C	T	C	G
Ireland--2009	1772_852_4	C	G	A	C	G	TC	C	T	CT	G
Ireland--2009	1772_852_40	C	A	A	C	G	TC	C	T	CT	G
Ireland--2009	1772_852_41	C	G	G	CT	G	T	CT	T	C	
Ireland--2009	1772_852_42	T	G	G	CT	G	TC	C	T	T	
Ireland-2009	1772_852_43	C	GA	GA	CT	G	TC	CT	T	C	G
Ireland-2009	1772_852_44	C	G	A	CT	GA	C	C	T	CT	G
Ireland--2009	1772_852_45	CT	G	A	T	A	TC	C	T	CT	G
Ireland--2009	1772_852_46	C	G	GA	T	G	TC	T	T	C	G
Ireland--2009	1772_852_47	C	G	GA	T	G	C	CT	T	T	G
Ireland--2009	1772_852_9	C	GA	GA	CT	G	C	C	T	C	G
Ireland--2009	1773_853_48	C	GA	A		G	TC	CT	A	C	A
Ireland--2009	1773_853_49	C	A	A	C	G	TC	CT	TA	C	
Ireland--2009	1773_853_50	C	A	A	T		C	C	TA	C	
Ireland--2009	1773_853_51	C	G	A	CT	G	TC	C	TA	CT	G
Ireland--2009	1773_853_52	C	G	A	T	G	C	C	TA	C	G
Ireland--2009	1773_853_53	C	G	GA	CT	G	C	C	TA	C	G
Ireland--2009	1773_853_54	C	G	A	CT	G	TC	C	TA	C	A
Ireland--2009	1773_853_55	C	G	A		G	C	CT	A	C	G
Ireland--2009	1773_853_56	C	GA	A		A	TC	CT	A	C	
Ireland--2009	1773_853_57	C	G	A	CT	G	TC	CT	TA	C	G
Ireland--2009	1773_853_58	C	G	A		G	TC	CT	A	C	
Ireland--2009	1773_853_59	C	G	A	C	A	C	T	A	C	A
Ireland--2009	1773_853_60	C	G	A	C	G	C	T	A	C	A
Ireland--2009	1773_853_61	C	GA	A	CT	A	TC	C	A	C	G
Ireland--2009	1773_853_62	C	A	A	CT		TC	C	TA	C	G
Ireland--2009	1773_853_63	C	A	A	T	A	C	C	TA	C	A
Ireland--2009	1773_853_64	C	GA	GA	CT	G	TC	CT	TA	CT	
Ireland--2009	1773_853_65	C	G	A	CT	G	C	C	A	C	A
Ireland--2009	1773_853_66	C	GA	A	C	G	TC	CT	TA	C	
Ireland--2009	1773_853_67	C	GA	A	CT	G	TC	C	A	C	
Ireland--2009	1773_853_68	C	GA	A	CT	A	TC	C	TA	C	
Ireland--2009	1773_853_69	C	GA	A	CT	A	TC	C	A	C	A
Ireland--2009	1773_853_70	C	G	A	CT	G	TC	C	TA	CT	G

Ireland--2009	1773_853_71	C	A	A	T	G	C	C	A	C	
Ireland--2009	1773_853_72	C	G	A		G	TC	CT	A	C	
Ireland--2009	1773_853_73	CT	A	A	CT	G	TC	C	TA	CT	
Ireland--2009	1773_853_74	C	GA	A	CT	G	TC	C	TA	C	
Ireland-2009	1773_853_75	C	G	A		G	TC	CT	A	C	G
Ireland--2009	1773_853_76	C	G	A	T	G	C	C	TA	C	
Ireland--2009	1773_853_77	C	G	A		G	TC	CT	A	C	A
Ireland--2009	1773_853_78	C	G	A	CT	A	TC	C	TA	CT	G
Ireland--2009	1773_853_79	C	GA	A	CT	G	TC	CT	A	C	G
Ireland--2009	1773_853_80	C	GA	A	CT	A	TC	C	TA	C	A
Ireland--2009	1773_853_81	C	G	A	CT	G	C	CT	A	C	A
Ireland--2009	1773_853_82	C	GA	A		G	TC	CT	A	C	
Ireland--2009	1773_853_83	C	GA	GA		G	TC	T	TA	C	
Ireland--2009	1773_853_84	C	G	A	CT	G	C	CT	A	C	
Ireland--2009	1773_853_85	C	G	A	CT	G	TC	C	TA	C	G
Ireland--2009	1773_853_86	C	GA	A		G	TC	CT	TA	CT	G
Ireland--2009	1773_853_87	C	G	A	CT	A	TC	C	TA	C	A
Ireland--2009	1773_853_88	CT	GA	A	CT	A	TC	C	TA	C	
Ireland--2009	1773_853_89	C	G	A	T	G	TC	C	A	C	G
Ireland--2009	1773_853_90	C	GA	A	CT	A	TC	CT	TA	CT	G
Ireland-2009	1773_853_91	C	G	A	CT	G	C	C	A	C	G
Ireland--2009	1773_853_92	CT	A	A	CT	G	TC	C	A	CT	G
Ireland--2009	1773_853_93	C	GA	GA	C	G	TC	CT	TA	C	
Ireland--2009	1773_853_94	C	GA	A	CT	A	TC	CT	A	C	A
Canada-2009_a	1774_857_1	CT	G	GA	CT	G	TC	C	TA	CT	
Canada-2009_a	1774_857_10	C	GA	A	CT	G	C	C	TA	C	G
Canada-2009_a	1774_857_11	C	G	GA	C	G	TC	C	T	C	G
Canada-2009_a	1774_857_12	C	G	A	C	G	TC	C	T	CT	
Canada-2009_a	1774_857_13	C	G	G	C	G	TC	C	T	C	G
Canada-2009_a	1774_857_14	CT	G	A	C	G	C	C	T	T	G
Canada-2009_a	1774_857_15	C	G	A	C	G	C	C	T	C	
Canada-2009_a	1774_857_16	C	G	A	C	G	C	C	T	CT	G
Canada-2009_a	1774_857_17	C	GA	GA	C	A	T	CT	T	T	G
Canada-2009_a	1774_857_18	C	GA	G	T	G	C	C	T	C	G
Canada-2009_a	1774_857_19	C	G	GA	C	A	T	C	T	CT	G
Canada-2009_a	1774_857_2	CT	G	GA	C	G	TC	CT	TA	C	G
Canada-2009_a	1774_857_20	C	GA	G	CT	G	T	T	T	T	
Canada-2009_a	1774_857_21	C	G	A	T	A	C	C	T	CT	G
Canada-2009_a	1774_857_22	C	GA	GA	C	G	TC	C	T	C	G

Canada-2009_a	1774_857_23	CT	G	GA	CT	G	C	C	TA	C	
Canada-2009_a	1774_857_24	CT	G	GA	T	A	TC	C	TA	C	G
Canada-2009_a	1774_857_25	C	GA	G	C	G	TC	C	T	CT	
Canada-2009_a	1774_857_26	C	G	A	C	G	T	C	TA	T	
Canada-2009_a	1774_857_27	C	G	GA	CT	A	C	CT	T	C	
Canada-2009_a	1774_857_28	CT	G	GA	C	G	TC	C	TA	C	G
Canada-2009_a	1774_857_29	CT	G	GA	CT	G	C	C	TA	CT	
Canada-2009_a	1774_857_3	CT	G	GA	C	G	TC	C	TA	C	
Canada-2009_a	1774_857_30	C	G	GA	CT	G	T	C	T	CT	A
Canada-2009_a	1774_857_31	CT	G	GA	CT	G	TC	C	TA	C	A
Canada-2009_a	1774_857_32	CT	G	A	C	G	C	CT	TA	CT	G
Canada-2009_a	1774_857_33	C	G	G	C	G	TC	CT	T	C	G
Canada-2009_a	1774_857_34	CT	G	A	C	G	C	CT	TA	C	G
Canada-2009_a	1774_857_35	T	G	GA	C	G	TC	C	T	C	G
Canada-2009_a	1774_857_36	T	G	A	C	G	C	CT	T	C	G
Canada-2009_a	1774_857_37	CT	G	GA	CT	G	C	C	T	CT	G
Canada-2009_a	1774_857_38	C	GA	GA		G	C	CT	TA	C	G
Canada-2009_a	1774_857_39	CT	G	GA	CT	G	TC	CT	T	T	G
Canada-2009_a	1774_857_4	CT	G	GA	C	G	C	CT	T	C	G
Canada-2009_a	1774_857_40	C	G	GA	C	G	TC	CT	T	CT	G
Canada-2009_a	1774_857_41	C	G	G	CT	A	TC	C	T	CT	G
Canada-2009_a	1774_857_42	C	A	GA	C	G	C	C	T	T	G
Canada-2009_a	1774_857_43	C	G	GA	CT	G	TC	C	T	CT	G
Canada-2009_a	1774_857_44	C	G	GA	C	G	T	C	T	T	
Canada-2009_a	1774_857_45	C	G	GA	CT	G	TC	C	TA	CT	G
Canada-2009_a	1774_857_46	CT	GA	GA	CT	G	C	C	TA	CT	G
Canada-2009_a	1774_857_47	T	G	A	CT	G	TC	C	T	C	G
Canada-2009_a	1774_857_5	CT	G	A	C	G	C	CT	TA	C	A
Canada-2009_a	1774_857_6	C	G	GA	C	G	C	CT	T	C	
Canada-2009_a	1774_857_7	CT	G	A	CT	G	TC	C	T	C	G
Canada-2009_a	1774_857_8	CT	G	G	CT	G	C	C	T	C	
Canada-2009_a	1774_857_9	C	G	GA	CT		T	CT	T	CT	G
Canada-2009_b	1775_858_48	C	G	GA	T	G	C	CT	TA	CT	
Canada-2009_b	1775_858_49	C	A	GA	CT	A	C	C	TA	CT	G
Canada-2009_b	1775_858_50	CT	G	GA	CT	G	C	CT	TA	CT	G
Canada-2009_b	1775_858_51	C	GA	GA	C	G	T	C	T	T	
Canada-2009_b	1775_858_52	C	G	GA	C	G	TC	CT	TA	C	
Canada-2009_b	1775_858_53	CT	G	A		A	TC	T	TA	T	G
Canada-2009_b	1775_858_54	CT	G	G	C	G	C	CT	T	C	G

Canada-2009_b	1775_858_55	CT	GA	GA	CT	G	T	CT	T	C	G
Canada-2009_b	1775_858_56	C	G	GA	T	A	TC	C	T	CT	G
Canada-2009_b	1775_858_57	C	GA	G	CT	G	C	CT	T	C	G
Canada-2009_b	1775_858_58	CT	G	GA	CT	G	C	C	TA	C	G
Canada-2009_b	1775_858_59	CT	GA	GA	CT	G	C	C	TA	CT	G
Canada-2009_b	1775_858_60	C	GA	G	C	A	C	C	T	CT	
Canada-2009_b	1775_858_61	CT	G	A	CT	A	TC	C	T	C	A
Canada-2009_b	1775_858_62	CT	G	A	C	A	C	C	T	C	G
Canada-2009_b	1775_858_63	C	G	GA	CT	G	TC	CT	T	CT	G
Canada-2009_b	1775_858_64	C	GA	GA	C	G	C	CT	T	C	G
Canada-2009_b	1775_858_65	C	G	G	CT	A	C	CT	T	C	G
Canada-2009_b	1775_858_66	C	G	GA	CT	G	TC	CT	T	C	
Canada-2009_b	1775_858_67	C	G	GA	CT	G	C	C	T	C	G
Canada-2009_b	1775_858_68	CT	G	GA		G	C	CT	TA	T	
Canada-2009_b	1775_858_69	C	G	GA	CT	G	TC	CT	TA	CT	A
Canada-2009_b	1775_858_70	CT	GA	GA	CT	A	TC	C	T	CT	A
Canada-2009_b	1775_858_71	C	G	A	CT	G	C	C	TA	C	G
Canada-2009_b	1775_858_72	C	G	GA	C	G	TC	C	T	C	G
Canada-2009_b	1775_858_73	C	G	A	C	G	TC	C	T	CT	G
Canada-2009_b	1775_858_74	C	G	GA	C	G	C	T	T	CT	
Canada-2009_b	1775_858_75	C	G	G	CT	G	TC	CT	T	C	
Canada-2009_b	1775_858_76	C	GA	G		G	TC	T	TA	C	G
Canada-2009_b	1775_858_77	C	G	A	C	A	TC	C	T	T	G
Canada-2009_b	1775_858_78	C	G	A	CT	G	TC	CT	T	CT	G
Canada-2009_b	1775_858_79	C	G	GA	C	G	TC	CT	TA	C	G
Canada-2009_b	1775_858_80	C	G	A	CT	G	C	C	TA	CT	G
Canada-2009_b	1775_858_81	C	G	A	CT	A	C	C	T	C	G
Canada-2009_b	1775_858_82	C	G	GA	CT	G	TC	CT	T	C	
Canada-2009_b	1775_858_83	C	GA	GA	C	G	T	C	T	T	G
Canada-2009_b	1775_858_84	C	GA	A	CT	G	C	C	TA	C	G
Canada-2009_b	1775_858_85	C	GA	G	T	G	T	CT	T	C	
Canada-2009_b	1775_858_86	C	G	GA	CT	G	TC	CT	T	C	G
Canada-2009_b	1775_858_87	C	G	A	CT	A	TC	C	T	C	G
Canada-2009_b	1775_858_88	C	G	GA	CT	A	C	C	TA	CT	
Canada-2009_b	1775_858_89	C	GA	GA	T	A	TC	C	TA	C	
Canada-2009_b	1775_858_90	C	GA	G	T	G	TC	T	T	C	G
Canada-2009_b	1775_858_91	C	G	G	C	A	TC	C	T	T	G
Canada-2009_b	1775_858_92	CT	G	G	C	A	TC	C	T	C	G
Canada-2009_b	1775_858_93	T	G	A	C	G	C	CT	T	C	

Canada-2009_b	1775_858_94	CT	G	G	C	G	C	C	T	CT	G
Faroe islands-2009	1768_850_1	C	GA	A	C	G	C	C	T	CT	
Faroe islands-2009	1768_850_10	C	GA	GA	CT	G	T	CT	T	CT	
Faroe islands-2009	1768_850_11	CT	GA	GA	T	G	TC	CT	T	C	
Faroe islands-2009	1768_850_12	C	GA	A	CT	G	C	C	T	C	G
Faroe islands-2009	1768_850_13	C	G	A	CT	G	T	CT	T	CT	
Faroe islands-2009	1768_850_14	C	G	GA	CT	G	C	C	T	C	
Faroe islands-2009	1768_850_15	C	G	A	CT	G	C	C	T	CT	G
Faroe islands-2009	1768_850_16	C	GA	A	C	G	C	CT	T	T	G
Faroe islands-2009	1768_850_17	CT	G	GA	CT	G	T	CT	T	C	G
Faroe islands-2009	1768_850_18	C	G	GA	C	A	TC	C	T	C	
Faroe islands-2009	1768_850_19	C	G	A	C	G	T	C	T	C	G
Faroe islands-2009	1768_850_2	C	G	A	CT	G	TC	C	T	CT	
Faroe islands-2009	1768_850_20	C	G	GA	T	G	C	C	T	C	G
Faroe islands-2009	1768_850_21	CT	G	A	T	A	C	C	T	C	
Faroe islands-2009	1768_850_22	C	G	A	C	G	C	C	T	C	
Faroe islands-2009	1768_850_23	C	G	GA	C	G	T	C	T	C	
Faroe islands-2009	1768_850_24	C	GA	G	CT	G	TC	CT	T	CT	G
Faroe islands-2009	1768_850_25	C	G	A	CT	G	TC	CT	T	CT	G
Faroe islands-2009	1768_850_26	T	G	A	CT	G	TC	C	T	CT	
Faroe islands-2009	1768_850_27	C	GA	G	T	G	T	T	T	C	G
Faroe islands-2009	1768_850_28	C	G	A	T	G	C	C	T	CT	
Faroe islands-2009	1768_850_29	C	G	A	C	G	TC	C	T	C	G
Faroe islands-2009	1768_850_3	C	G	A	T	A	C	C	T	C	
Faroe islands-2009	1768_850_30	C	G	A	CT	G	TC	C	T	CT	
Faroe islands-2009	1768_850_31	C	G	A	C	G	T	C	T	T	
Faroe islands-2009	1768_850_32	C	G	GA	CT	G	T	CT	T	C	
Faroe islands-2009	1768_850_33	CT	GA	G	CT	G	TC	C	T	C	
Faroe islands-2009	1768_850_34	C	G	A	CT	G	TC	C	T	C	G
Faroe islands-2009	1768_850_35	C	G	A	C	G	T	C	T	T	G
Faroe islands-2009	1768_850_36	C	G	GA	T	G	T	T	T	C	
Faroe islands-2009	1768_850_37	C	G	A	CT	A	C	C	T	C	G
Faroe islands-2009	1768_850_38	C	G	A	CT	A	TC	C	T	C	
Faroe islands-2009	1768_850_39	C	GA	GA	CT	G	T	CT	T	C	G
Faroe islands-2009	1768_850_4	C	GA	GA	CT	G	C	C	T	CT	
Faroe islands-2009	1768_850_40	C	GA	A		G		C	T		
Faroe islands-2009	1768_850_41	C	G	A	C	G	T	C	T	C	
Faroe islands-2009	1768_850_42	C	G	A	CT	A	TC	C	T	C	
Faroe islands-2009	1768_850_43	C	GA	GA	T	G	TC	CT	T	C	

Faroe islands-2009	1768_850_44	C	G	A	CT	G	C	C	T	T	A
Faroe islands-2009	1768_850_45	C	G	A	CT	G	T	C	T	CT	G
Faroe islands-2009	1768_850_46	C	GA	GA	CT	G	TC	C	T	CT	G
Faroe islands-2009	1768_850_47	C	G	A	C	G	T	C	T	CT	
Faroe islands-2009	1768_850_5	CT	A	GA	C	G	T	CT	T	CT	
Faroe islands-2009	1768_850_6	C	G	A	CT	A	TC	C	T	C	
Faroe islands-2009	1768_850_7	C	G	A	C	G	T	C	T	T	G
Faroe islands-2009	1768_850_8	C	GA	A	CT	G	TC	C	T	CT	G
Faroe islands-2009	1768_850_9	C	G	G	T	G	T	T	T	C	G
Faroe islands-2009	1769_851_1	C	G	GA	CT	G	TC	C	T	T	
Faroe islands-2009	1769_851_10	C	GA	GA	CT	G	T	CT	T	CT	
Faroe islands-2009	1769_851_11	C	G	GA	CT	G	T	C	T	CT	A
Faroe islands-2009	1769_851_12	C	GA	GA	C	G	T	CT	T	C	G
Faroe islands-2009	1769_851_13	C	G	A	C	G	TC	C	T	CT	
Faroe islands-2009	1769_851_14	C	G	GA	CT	G	T	C	T	CT	
Faroe islands-2009	1769_851_15	C	GA	GA	CT	G	TC	C	T	CT	
Faroe islands-2009	1769_851_16	C	G	A		A	C	CT	T	CT	
Faroe islands-2009	1769_851_17	C	GA	A	C	G	TC	CT	TA	C	A
Faroe islands-2009	1769_851_18	C	G	A	C	G	TC	C	T	C	
Faroe islands-2009	1769_851_19	C	G	GA	CT	G	C	C	T	CT	
Faroe islands-2009	1769_851_2	C	G	G	CT	G	C	CT	T	C	G
Faroe islands-2009	1769_851_20	C	G	A	CT	G	TC	CT	TA	C	
Faroe islands-2009	1769_851_21	C	G	GA	CT	G	TC	CT	T	T	
Faroe islands-2009	1769_851_22	CT	G	A	CT	A	C	C	T	C	
Faroe islands-2009	1769_851_23	C	G	A	CT	G	TC	CT	T	CT	
Faroe islands-2009	1769_851_24	C	G	G	CT	G	TC	CT	T	CT	G
Faroe islands-2009	1769_851_25	C	G	A	CT	A	T	C	T	T	G
Faroe islands-2009	1769_851_26	C	GA	GA	CT	G	C	CT	T	C	
Faroe islands-2009	1769_851_27	C	G	G	C	G	T	C	T	C	G
Faroe islands-2009	1769_851_28	C	G	A	T	A	TC	CT	T	CT	
Faroe islands-2009	1769_851_29	C	GA	GA		G	TC	CT	TA	C	G
Faroe islands-2009	1769_851_3	C	G	GA	CT	G	TC	C	T	T	
Faroe islands-2009	1769_851_30	C	G	A	CT	G	TC	C	T	CT	
Faroe islands-2009	1769_851_31	CT	G	GA	CT	G	T	CT	T	CT	A
Faroe islands-2009	1769_851_32	C	GA	GA	T	G	C	C	T	C	
Faroe islands-2009	1769_851_33	C	GA	GA	CT	G	TC	CT	T	C	
Faroe islands-2009	1769_851_34	CT	A	A	CT	G	TC	C	T	C	
Faroe islands-2009	1769_851_35	C	A	G	CT	G	T	CT	T	CT	A
Faroe islands-2009	1769_851_36	CT	G	A	C	G	TC	CT	TA	T	G

Faroe islands-2009	1769_851_37	C	G	A	C	G	TC	C	T	C	
Faroe islands-2009	1769_851_38	C	GA	G	CT	G	T	CT	T	CT	
Faroe islands-2009	1769_851_39	C	G	A		G	TC	CT	TA	T	
Faroe islands-2009	1769_851_4	C	G	A	C	G	T	C	T	T	G
Faroe islands-2009	1769_851_40	C	G	A	CT	G	TC	C	T	CT	
Faroe islands-2009	1769_851_41	C	G	GA	T	G	TC	CT	T		A
Faroe islands-2009	1769_851_42	C	GA	GA	T	G	TC	CT	T	C	G
Faroe islands-2009	1769_851_43	CT	A	GA	C	G	TC	CT	T	CT	
Faroe islands-2009	1769_851_44	C	G	GA	CT	G	TC	C	T	CT	G
Faroe islands-2009	1769_851_45	C	GA	GA	C	G	T	C	T	C	
Faroe islands-2009	1769_851_46	C	G	A	CT	G	TC	C	T	C	
Faroe islands-2009	1769_851_47					G		CT	T		
Faroe islands-2009	1769_851_5	C	G	A	CT	G	TC	C	T	C	
Faroe islands-2009	1769_851_6	CT	GA	GA	CT	G	T	CT	T	T	A
Faroe islands-2009	1769_851_7	C	G	A	C	G	T	C	T	CT	
Faroe islands-2009	1769_851_8	C	G	A	T	A	C	C	T	T	G
Faroe islands-2009	1769_851_9	C	G	A	CT	G	TC	C	T	T	G
Shetland-2009	1770_855_1	C	GA	GA	T	A	TC	CT	T	CT	G
Shetland-2009	1770_855_10	C	G	A	C	G	T	C	T	T	
Shetland-2009	1770_855_11	C	GA	GA	CT	GA	TC	CT	T	CT	
Shetland-2009	1770_855_12	CT	GA	G	CT	G	TC	CT	T	T	
Shetland-2009	1770_855_13	C	G	A	CT	A	TC	C	T	CT	
Shetland-2009	1770_855_14	C	G	A	CT	A	TC	C	T	C	G
Shetland-2009	1770_855_15										
Shetland-2009	1770_855_16	CT			T	A	TC	C	T	CT	
Shetland-2009	1770_855_17	C	G	A	C	A	C	C	T	C	
Shetland-2009	1770_855_18	C	G	A	T	A	C	C	T	C	G
Shetland-2009	1770_855_19	C	G	A	T	A	C	C	T	C	
Shetland-2009	1770_855_2	CT	G	A	T	GA	C	C	TA	CT	G
Shetland-2009	1770_855_20	C	G	A	C	G	TC	C	T	CT	
Shetland-2009	1770_855_21	C	G	A	CT	A	TC	C	T	CT	G
Shetland-2009	1770_855_22	C	G	A	CT	A	T	CT	T	CT	G
Shetland-2009	1770_855_23	C	GA	A		G		C	T	T	G
Shetland-2009	1770_855_24	C	GA	GA	CT	G	T	CT	T	C	G
Shetland-2009	1770_855_25	C	G	A	T	A	C	C	T	T	
Shetland-2009	1770_855_26	C	G	GA	C	G	C	C	T	T	
Shetland-2009	1770_855_27	C	G	A	CT	A	TC	C	T	CT	
Shetland-2009	1770_855_28	C	G	A	T	A	TC	CT	T	CT	G
Shetland-2009	1770_855_29	C	G	A	T	G	C	C	TA	CT	G

Shetland-2009	1770_855_3	C	G	A	C	G		C	TA	CT	G
Shetland-2009	1770_855_30	C	G	A	CT	G	TC	C	T	T	
Shetland-2009	1770_855_31	C	G	GA	C	G	TC	C	T	CT	G
Shetland-2009	1770_855_32	C	G	A	CT	G	TC	C	T	CT	G
Shetland-2009	1770_855_33	CT	G	A	CT	G	C	C	T	CT	G
Shetland-2009	1770_855_34	C	G	GA	T	G	TC	CT	T	C	G
Shetland-2009	1770_855_35	C	G	A	CT	A	TC	CT	T	C	
Shetland-2009	1770_855_36	C	G	A	T	A	C	C	T	C	
Shetland-2009	1770_855_37	CT	G	A	T	A	TC	C	T	CT	G
Shetland-2009	1770_855_38	C	G	A	T	A	C	C	T	C	A
Shetland-2009	1770_855_39	C	GA	A	C	G	TC	CT	TA	C	
Shetland-2009	1770_855_4	C	G	A		GA		CT	TA	C	G
Shetland-2009	1770_855_40	C	G	GA	T	A	TC	C	T	T	
Shetland-2009	1770_855_41	C	G	A	T	A	TC	C	T	T	G
Shetland-2009	1770_855_42	CT	GA	GA	CT	G	T	CT	T	T	A
Shetland-2009	1770_855_43	CT	GA	GA	T	G	TC	CT	T	T	
Shetland-2009	1770_855_44			A		G		C	T		G
Shetland-2009	1770_855_45	C	G	GA	T		TC	CT	T	C	
Shetland-2009	1770_855_46	C	G	G	T	A	T	T	T	T	
Shetland-2009	1770_855_47	C	G	A	C	A	TC	C	T	CT	
Shetland-2009	1770_855_5	C	G	A	C	A	C	C	T	C	A
Shetland-2009	1770_855_6	C	G	A	T	G	T	C	T	T	G
Shetland-2009	1770_855_7	C	GA	A	C	A	TC	CT	TA	T	G
Shetland-2009	1770_855_8	C	G	A	T	A	C	CT	T	T	
Shetland-2009	1770_855_9	C	G	A	C	G	T	C	T	C	
Shetland-2009	1771_856_1	C	G	A	CT	A	TC	C	T	CT	
Shetland-2009	1771_856_10	C	G	A	CT	A	TC	C	T	CT	G
Shetland-2009	1771_856_11	C	G	A	T	A	C	C	T	CT	
Shetland-2009	1771_856_12	C	GA	A	C	A	TC	CT	T	CT	
Shetland-2009	1771_856_13	C	GA	A	CT	G	T	C	T	C	
Shetland-2009	1771_856_14	C	G	A	CT	A	TC	C	T	CT	A
Shetland-2009	1771_856_15	C	G	A	T	A	C	C	T	C	G
Shetland-2009	1771_856_16	C	G	A	C	A	TC	C	T	C	A
Shetland-2009	1771_856_17	C	G	A	CT	A	T	C	T	CT	G
Shetland-2009	1771_856_18	C	G	A	C	G	T	C	T	T	
Shetland-2009	1771_856_19	C	A	GA	T	G	TC	CT	T	C	A
Shetland-2009	1771_856_2	C	G	A	T	G	C	CT	T	C	
Shetland-2009	1771_856_20	C	G	A	T	G	C	C	TA	C	
Shetland-2009	1771_856_21	C	G	A	CT	G	TC	C	T	T	

Shetland-2009	1771_856_22	C	GA	A	CT	A	TC	C	TA	CT	
Shetland-2009	1771_856_23	C	G	GA	CT	G	C	C	T	C	
Shetland-2009	1771_856_24	C	GA	A	C	A	T	C	T	CT	
Shetland-2009	1771_856_25	C	GA	GA	T	G	C	C	T	C	
Shetland-2009	1771_856_26	C	GA	A	C	G	C	T	TA	CT	
Shetland-2009	1771_856_27	C	G	A	C	G	T	C	T	T	G
Shetland-2009	1771_856_28	C	G	A	C	G	T	C	T	T	A
Shetland-2009	1771_856_29	C	G	A	CT	A	TC	C	T	CT	
Shetland-2009	1771_856_3	CT	G	A	C	A	T	C	T	T	
Shetland-2009	1771_856_30	C	G	A	C	A	T	C	T	T	
Shetland-2009	1771_856_31	C	G	A	C	A	TC	C	T	T	
Shetland-2009	1771_856_32	C	G	GA	T	A	TC	CT	T	C	G
Shetland-2009	1771_856_33	C	GA	GA	C	G	TC	CT	TA	CT	A
Shetland-2009	1771_856_34	C	G	A	CT	G	TC	C	T	CT	
Shetland-2009	1771_856_35	CT	G	A	CT	G	TC	C	T	CT	
Shetland-2009	1771_856_36	C	GA	A	T	GA	C	C	T	CT	
Shetland-2009	1771_856_37	C	GA	A		G	C	CT	A	CT	G
Shetland-2009	1771_856_38	C	GA	A	T	A	C	C	T	C	
Shetland-2009	1771_856_39	C	GA	A	C	G	TC	CT	T	C	
Shetland-2009	1771_856_4	C	GA	A	CT	G	TC	C	T	T	G
Shetland-2009	1771_856_40	C	G	A		A	C	CT	T	CT	G
Shetland-2009	1771_856_41	C	G	A	CT	A	TC	C	T	C	G
Shetland-2009	1771_856_42	C	GA	A	T	A	TC	C	T	CT	A
Shetland-2009	1771_856_43	C	G	A	T	G	C	C	T	C	
Shetland-2009	1771_856_44	C	G	A	CT	A	TC	C	T	C	
Shetland-2009	1771_856_45	C	GA	GA	CT	G	T	CT	T	T	G
Shetland-2009	1771_856_46	C	GA	A	CT	G	TC	C	T	CT	G
Shetland-2009	1771_856_47	C	G	A	CT	A	TC	C	T	T	A
Shetland-2009	1771_856_5	CT	GA	A	C	A	T	C	T	CT	
Shetland-2009	1771_856_6	C	G	A	CT	G	T	C	T	T	
Shetland-2009	1771_856_7	C	GA	A	C	G	T	C	T	T	G
Shetland-2009	1771_856_8	C	G	A	CT	G	TC	C	T	CT	G
Shetland-2009	1771_856_9	C	G	A	CT	G	TC	C	T	CT	G
Ireland-2009	1772_852_5	T	GA	GA	CT	G	T	T	T	CT	
Ireland-2009	1772_852_6	CT	G	A	C	G	C	C	T	T	G
Ireland-2009	1772_852_7	C	G	GA	T	G	TC	C	T	CT	G
Ireland-2009	1772_852_8	C	A	A	T	G	TC	T	T	C	
Canada-2002	LsC1	C	G	GA	C	G	TC	CT	T	T	
Canada-2002	LsC10	C	GA	G	C	A	C	CT	T	T	

Canada-2002	LsC11	C	GA	GA	CT	G	T	C	T	CT	G
Canada-2002	LsC12	C	G	GA	CT	G	C	C	TA	CT	G
Canada-2002	LsC13	C	G	GA	C	A	TC	C	T	C	G
Canada-2002	LsC14	C	G	GA	C	G	TC	CT	TA	C	G
Canada-2002	LsC15	C	G	GA	C	G	TC	C	T	CT	G
Canada-2002	LsC16	C	G	G	CT		T	C	T	C	G
Canada-2002	LsC17	C	A	A	CT	G	C	CT	T	C	G
Canada-2002	LsC18	CT	G	GA	C	G	C	C	T	C	
Canada-2002	LsC19	CT	G	GA	C	G	C	CT	TA	C	G
Canada-2002	LsC2	C	G	GA	T	G	TC	CT	T	T	
Canada-2002	LsC20	C	G	GA	C	G	C	C	T	C	G
Canada-2002	LsC21	C	G	GA	C	G	C	C	T	C	G
Canada-2002	LsC22	C	G	GA	CT	G	C	C	T	CT	G
Canada-2002	LsC23	CT	G	GA	T		C	C	TA	C	G
Canada-2002	LsC24	CT	G	G	CT		C	C	T	CT	G
Canada-2002	LsC25	CT	GA	G	C	GA	C	CT	T	T	G
Canada-2002	LsC26	CT	G	GA	C		T	C	T	CT	G
Canada-2002	LsC27	C	G	GA	C	G	T	T	TA	CT	G
Canada-2002	LsC28	C	A	A	CT	G	C	CT	T	C	G
Canada-2002	LsC29	C	G	A	C	G	T	T	TA	CT	G
Canada-2002	LsC3	C	GA	G	CT	A	TC	C	T	C	G
Canada-2002	LsC30	C	GA	GA	CT	G	T	C	T	T	G
Canada-2002	LsC4	CT	G	G	CT	A	TC	C	T	CT	G
Canada-2002	LsC5	C	G	GA	CT	A	TC	C	T	C	G
Canada-2002	LsC6	C	GA	A	CT	G	TC	C	TA	CT	G
Canada-2002	LsC7	C	G	A	C	G	TC	C	T	C	
Canada-2002	LsC8	C	G	G	C	G	TC	C	T	T	G
Canada-2002	LsC9	C	G	GA	CT	G	C	C	T	T	G
Scotland-2002_b	LsK1	C	G	A	CT	G	TC	C	TA	C	G
Scotland-2002_b	LsK10	CT	GA	GA	T	A	TC	C	A	C	
Scotland-2002_b	LsK11	C	G	A	CT	G	TC	C	TA	C	
Scotland-2002_b	LsK12	C	G	A	CT	G		C	A	CT	
Scotland-2002_b	LsK13	C	G	A	CT	G		C	A	C	
Scotland-2002_b	LsK14	CT	G	A	T	G	C	C	TA	C	
Scotland-2002_b	LsK15	C	G	A	T	G	C	C	TA	C	
Scotland-2002_b	LsK16	C	GA	A	CT	G	TC	C	TA	CT	G
Scotland-2002_b	LsK17	CT	G	A	T	G	TC	C	TA	CT	A
Scotland-2002_b	LsK18	C	G	A	CT	G	C	C	TA	C	
Scotland-2002_b	LsK19	C	G	A	CT	G	TC	C	A	C	G

Scotland-2002_b	LsK2	C	G	A	T	A	TC	C	TA	CT	
Scotland-2002_b	LsK20	C	GA	A	CT	G	TC	C	TA	CT	G
Scotland-2002_b	LsK21	C	G	GA	CT	G	C	C	TA	CT	G
Scotland-2002_b	LsK22	C	G	A	CT	G	TC	C	A	C	G
Scotland-2002_b	LsK23	C	G	A	T	G	C	C	TA	C	G
Scotland-2002_b	LsK24	C	G	A	CT	A	TC	C	A	C	G
Scotland-2002_b	LsK25	C	G	A	T	G	C	C	TA	C	G
Scotland-2002_b	LsK26	CT	G	A	T	G	C	C	TA	CT	G
Scotland-2002_b	LsK27	C	G	A		G	TC	C	TA	C	G
Scotland-2002_b	LsK28	CT	GA	GA	CT	G	TC	C	TA	CT	G
Scotland-2002_b	LsK29	C	G	A	CT	G	TC	C	TA	CT	G
Scotland-2002_b	LsK3	C	GA	A	CT	A	TC	C	TA	CT	G
Scotland-2002_b	LsK30	C	G	A	CT	G	TC	C	TA	C	G
Scotland-2002_b	LsK4	C	G	A	CT	G	TC	C	TA	CT	
Scotland-2002_b	LsK5	C	G	A	CT	G	TC	C	TA	C	G
Scotland-2002_b	LsK6	CT	G	A	CT	G	TC	C	A	C	G
Scotland-2002_b	LsK7	C	GA	A	T	G	TC	C	A	C	G
Scotland-2002_b	LsK8	C	G	A	CT	G	C	C	TA	C	G
Scotland-2002_b	LsK9	C	GA	A	CT	A	TC	C	A	C	
Norway-2002	LsSu1	T	G	A	CT	G	TC	CT	T	CT	G
Norway-2002	LsSu10	C	G	GA	T	G	TC	CT	T	CT	G
Norway-2002	LsSu11	C	G	G	C	A	TC	C	T	CT	G
Norway-2002	LsSu12	CT	GA	G	T	G	T	T	T	C	G
Norway-2002	LsSu13	C	G	G	C	G	TC	C	T	CT	G
Norway-2002	LsSu14	C	A	G	C	G	C	T	T	C	
Norway-2002	LsSu15	C	G	G	CT	G	C	CT	T	C	
Norway-2002	LsSu16	C	G	GA	C	G	C	CT	TA	C	G
Norway-2002	LsSu18	CT	GA	GA	CT	G	C	CT	T		
Norway-2002	LsSu19	C	GA	G	T	G	T	CT	T	C	G
Norway-2002	LsSu2	C	GA	G	CT	G	C	C	T	CT	
Norway-2002	LsSu20	T	G	GA	C	G	TC	CT	TA	C	G
Norway-2002	LsSu21	CT	A	G	CT	G	T	CT	T	C	G
Norway-2002	LsSu22	C	A	GA	CT	G	TC	T	T	C	G
Norway-2002	LsSu23	C	G	GA	C	G	TC	C	T	C	G
Norway-2002	LsSu24	CT	GA	GA		G	TC	T	TA	C	
Norway-2002	LsSu25	C	G	GA	C	G	TC	CT	TA	C	
Norway-2002	LsSu26	C	G	GA		G	C	CT	TA	C	G
Norway-2002	LsSu27	C	A	GA	CT	G	C	T	T	T	G
Norway-2002	LsSu28	C	GA	GA		G	C	CT	TA	C	G

Norway-2002	LsSu29	CT	GA	A	C	G	C	CT	TA	C	G
Norway-2002	LsSu3	C	GA	GA	CT	A	T	CT	T	CT	
Norway-2002	LsSu30	CT	G	GA		G	TC	T	TA	C	G
Norway-2002	LsSu4	C	GA	G	CT	G	TC	CT	T	C	
Norway-2002	LsSu5	C	GA	G	T	G	T	T	T	C	G
Norway-2002	LsSu6	CT	A	G	CT	G	TC	C	T	C	
Norway-2002	LsSu7	CT	GA	A		G	TC	T	TA	C	
Norway-2002	LsSu8	C	G	G	T	A	T	CT	T	C	
Norway-2002	LsSu9	CT	GA	G	CT	G	T	CT	T	C	G
Norway-2000	LsT1	C	G	GA	T	G	TC	CT	T	CT	
Norway-2000	LsT10	C	GA	GA	CT	A	C	CT	T	C	G
Norway-2000	LsT11	C	A	GA	T	G	T	T	T	C	G
Norway-2000	LsT12	CT	G	GA	C	G	TC	C	T	C	G
Norway-2000	LsT13	C	A	GA	C	A	TC	C	T	C	G
Norway-2000	LsT14	C	GA	GA	T	G	TC	CT	T	C	G
Norway-2000	LsT15	T	G	GA	CT	G	C	C	T	CT	G
Norway-2000	LsT16	C	G	GA	CT	G	C	CT	T	CT	G
Norway-2000	LsT17	CT		GA	CT	G		CT	T	C	G
Norway-2000	LsT18	C	G	GA	CT	G		C	T	CT	G
Norway-2000	LsT19	CT		GA	C	GA	TC	CT	TA	C	G
Norway-2000	LsT2	CT	GA	GA	C	A	C	CT	TA	C	G
Norway-2000	LsT20	C	GA	GA	C	GA	C	CT	T		G
Norway-2000	LsT21	CT	G	GA	CT	GA	TC	CT	T	CT	G
Norway-2000	LsT22	CT	G	A	T	G	C	T	T	C	G
Norway-2000	LsT23	C	GA	GA	C	G	T	C	T		G
Norway-2000	LsT24	C		GA	C	G		CT	TA	C	G
Norway-2000	LsT25	CT	GA	GA	CT	G	TC	CT	T	C	G
Norway-2000	LsT26	C	GA		CT	G	TC	CT	T	CT	G
Norway-2000	LsT27	CT	G	GA		G	C	CT	TA	CT	G
Norway-2000	LsT28	C	GA	GA		G	TC	CT	TA	C	G
Norway-2000	LsT29	C	GA	G	C	G		C	T	C	G
Norway-2000	LsT3	CT	G	GA	C	G	TC	C	T	CT	G
Norway-2000	LsT30	C		A	C	A	TC	C	T		G
Norway-2000	LsT4	C	G	GA	C	G	T	C	T	C	G
Norway-2000	LsT5	C	G	GA	C	G	TC	CT	TA	C	G
Norway-2000	LsT6	C	GA	GA	CT	G	C	C	T	C	G
Norway-2000	LsT7	C	G	GA	C	G	C	C	T	C	G
Norway-2000	LsT8	T	G	GA	C	G	TC	T	T	C	G
Norway-2000	LsT9	C	G	GA	CT	A	TC	CT	T	CT	G

Canada-1999	C1	CT	G		C	G	C	C	T	CT	G
Canada-1999	C10	CT	G	GA	CT	G	T	T	T	CT	G
Canada-1999	C11		GA			G	TC	CT	TA		G
Canada-1999	C12	T	G	A	CT	A	TC	CT	T	CT	G
Canada-1999	C13	C	G	A	C	G	C	CT	TA	C	
Canada-1999	C14	C	G	A		G	C	CT	TA	C	G
Canada-1999	C15	C	G	GA	C	GA	TC	C	T	CT	G
Canada-1999	C16	C	G	G	C	A	TC	CT	T	T	G
Canada-1999	C17	CT	G	A	CT	G	C	C	TA	CT	G
Canada-1999	C18	CT	G	GA	C	G	C	CT	TA	C	G
Canada-1999	C19	C	G	GA	CT	G	TC	C	T	C	A
Canada-1999	C2	C	GA	G	CT	G	T	CT	T	C	G
Canada-1999	C20	C	A	G	CT		TC	C	T	CT	G
Canada-1999	C21	C	G	G	C	G	C	CT	T	C	
Canada-1999	C22	C	GA	GA	CT	G	TC	C	TA	T	G
Canada-1999	C23				CT				T	CT	
Canada-1999	C24										
Canada-1999	C25	T	GA	GA	C	GA	TC	CT	T	C	G
Canada-1999	C26	C	GA	A	CT	G	TC	C	T	CT	G
Canada-1999	C27	C	G	GA	C	G	C	CT	TA	C	G
Canada-1999	C28	CT	GA	GA	C		TC	C	T	CT	G
Canada-1999	C29	CT		GA	C	GA	C	C	T	C	G
Canada-1999	C3	CT	GA	G	C	G	C	CT	T	C	G
Canada-1999	C30	C	G	GA	C	G		CT	TA	C	G
Canada-1999	C31			GA		G	TC	CT	T	C	G
Canada-1999	C32	C	G	GA	C	G	TC	C	T	CT	G
Canada-1999	C33	C	G	GA	C	A	C	C	T	CT	G
Canada-1999	C34	C	G	A	C	A	TC	C	T	C	
Canada-1999	C35	C	GA	A	CT	A	C	CT	T	C	
Canada-1999	C36	T	G	G	C	G	C	CT	T	C	G
Canada-1999	C37	CT	G		CT	GA	C	CT	T	CT	G
Canada-1999	C38	C	G	GA	CT	G	C	C	TA	CT	G
Canada-1999	C39	C	GA	G	CT	G	TC	CT	T	C	G
Canada-1999	C4	C	G	GA		G	TC	T	TA	C	G
Canada-1999	C40										
Canada-1999	C41	CT	G	G	CT	GA	TC	C	T	CT	A
Canada-1999	C42	C	G	A	C	GA	TC	C	T	C	G
Canada-1999	C43	C	G	GA	T	G	C	C	T	C	G
Canada-1999	C44	C	G	A	C	G	TC	C	TA	C	

Canada-1999	C45	C	G	GA	C	G	TC	C	T	CT	
Canada-1999	C46	C	G		CT	G	TC	CT	TA	C	G
Canada-1999	C47	T	GA	GA	C	GA	TC	CT	TA	C	G
Canada-1999	C48	CT	GA	A	C	G	TC	C	T	C	G
Canada-1999	C49	CT	G	GA	C	G	TC	C	T	T	
Canada-1999	C5	C	G	GA	C	G	TC	C	T	C	G
Canada-1999	C50	C	GA	GA	C	GA	TC	C	T	CT	G
Canada-1999	C51	C	G	GA	CT	G	C	C	T	T	
Canada-1999	C52	CT	GA		C		C	C	T	C	G
Canada-1999	C53	C	G	A	CT	G	C	C	T	C	G
Canada-1999	C54	C	G	GA	CT	G	C		TA	C	G
Canada-1999	C55	CT	G		CT	G	TC		TA		G
Canada-1999	C56	C	G	GA	CT	G	C				G
Canada-1999	C57	T	GA	A	C	G	TC	CT	T	CT	G
Canada-1999	C58	C	G	GA	C	G	TC	C	T	C	G
Canada-1999	C59	CT		GA		G	C	CT	TA	C	G
Canada-1999	C6	C	GA	A	C	G	TC	C	T	T	G
Canada-1999	C60	C	G	GA	C	G	C	CT	TA	C	G
Canada-1999	C61	C					C	CT	T	C	G
Canada-1999	C62	CT	G	G	C	G	C	CT	T	C	G
Canada-1999	C63	C	GA			G	TC		T	C	G
Canada-1999	C64										
Canada-1999	C65	C	G		CT		TC	CT	T	CT	G
Canada-1999	C66	CT	G	GA	C	A	C	CT	T	CT	G
Canada-1999	C67	C		GA	CT	G	T	CT	TA	CT	G
Canada-1999	C68	C	GA	A	C	G	T	CT	TA	C	G
Canada-1999	C69	C	GA	G	C	GA	C	CT	T	C	G
Canada-1999	C7	CT	G	GA	C	G	C	C	T	C	G
Canada-1999	C70	C	GA	G	CT	G	T	CT	T	C	G
Canada-1999	C71	C		GA	C	G			TA	C	G
Canada-1999	C72	C	G	GA	C	G	C	CT	TA	C	G
Canada-1999	C73	C		G		G	TC	CT	TA	C	
Canada-1999	C74	C	GA	G	T	G	TC	T	T	C	G
Canada-1999	C75	C	G	GA	CT	G	C	C	TA	CT	
Canada-1999	C76	C	G	A	CT	G	C	CT	TA	C	G
Canada-1999	C77	C	A	A	C	A	T	CT	T	CT	G
Canada-1999	C78	C	G	GA	C	G	TC	CT	T	CT	G
Canada-1999	C79	C	G	GA	C	G	TC	T	TA	C	G
Canada-1999	C8	C	GA	A		A	C	CT	A	C	G

Canada-1999	C80	CT	GA	A		G	TC	CT	A	C	G
Canada-1999	C81	C	G	GA	C	G	TC	C	T		
Canada-1999	C82	T	G	A	C	GA	TC	CT	TA	C	G
Canada-1999	C83	CT	G	GA	C	G	C	C	T	CT	G
Canada-1999	C84	CT	GA	GA	CT	G	C	CT	TA	C	G
Canada-1999	C85	C	G	GA	CT	G	TC	CT	T	C	G
Canada-1999	C86		G	GA	C	G		CT	TA	C	G
Canada-1999	C87	CT	G	GA	CT	G	TC	C	T	T	G
Canada-1999	C88	C	G	GA	T	G	C	C	T	T	
Canada-1999	C89	CT	GA	G	CT	GA	TC	C	T	CT	G
Canada-1999	C9	CT	G	G	CT	G	TC	T	T	T	G
Canada-1999	C90	C	GA	A	C	G	TC	C	T	C	G
Canada-1999	C91	C	G	G	C	G	C	C	T	C	G
Canada-1999	C92	CT	G	G	C	G	TC	C	T	CT	G
Canada-1999	C93	C	G	G	C		C	CT	TA	C	G
Canada-1999	C94	C	G	A	T	G	C	C	A	C	G
Canada-1999	C95	CT	G	GA	C	G	C	CT	T	C	G
Canada-1999	C96	C	G	GA	C	G		C	T		G
Ireland-2001	I1	C	G	A	T	G	TC	C	T	C	
Ireland-2001	I10	C	G	G	CT	A	TC	C	T	CT	A
Ireland-2001	I11	C	G	A	C	G	C	C	T	C	G
Ireland-2001	I12	C	G	GA	C	G	TC	CT	TA	CT	G
Ireland-2001	I13	C	GA	A	C	G	T	C	T	CT	G
Ireland-2001	I14	CT	G	GA	C	G	T	C	T	CT	G
Ireland-2001	I15	C	G	A	CT	G	TC	C	T	T	
Ireland-2001	I16	C	GA	A	C	G	T	C	T	CT	G
Ireland-2002	I17	C	G	A	C	G	TC	C	TA	CT	
Ireland-2002	I18	C	G	A	CT	G	TC	C	T		
Ireland-2002	I19	CT	GA	GA	T	A	C	C	T		
Ireland-2001	I2	C	G	GA	CT	GA	T	C	T	T	
Ireland-2002	I20	CT	A	GA	CT	A	C	C	T	T	G
Ireland-2002	I21	CT	G	A	T	G	C	C	T	C	
Ireland-2002	I22	CT	G	GA	CT	A	TC	CT	T	T	
Ireland-2002	I23	C	G	A	CT	G	C	CT	T	C	
Ireland-2002	I24	C	G	A	C	G	TC	C	T	C	G
Ireland-2002	I25	C	GA	GA	T	G	TC	C	T	CT	G
Ireland-2002	I26	C	GA	GA	T	G	TC	C	T	CT	G
Ireland-2002	I27	C	G	GA	CT		C	C	T	T	G
Ireland-2002	I28	C		GA	CT	G	TC	CT	T	C	G

Ireland-2002	I29	C	G	A	C	G	T	CT	T	T	
Ireland-2001	I3	T	GA	GA	C	G	TC	C	T	CT	G
Ireland-2002	I30	C	G	A	C	G	TC	C	T	T	
Ireland-2002	I31	CT	G	GA	CT		TC	C	T	CT	
Ireland-2002	I32	C	GA	A	CT	G	T	C	T	CT	G
Ireland-2001	I4			GA	C	G	C	CT	TA	C	G
Ireland-2001	I5	C	G	A	C	G	C	CT	TA	CT	G
Ireland-2001	I6	C	G	GA	T	G	TC	CT	T	C	G
Ireland-2001	I7	CT	G	A	C	G	T	C	TA	T	
Ireland-2001	I8	C	G	A	C	A	TC	C	T	C	G
Ireland-2001	I9	CT	G	A	T		T	CT	T	C	G
Norway-1998	N1	C		G	CT		C		T	CT	G
Norway-1998	N10	C	G	A	C	G	C	T	A	C	
Norway-1998	N100	C	G	GA		GA	C	T	TA	C	
Norway-1998	N101	CT	G	G	CT	G	TC	CT	T	C	
Norway-1998	N102	C	GA	GA	C	G	C	CT	TA	C	G
Norway-1998	N103	C	GA	GA	T	G	T	T	T	C	G
Norway-1998	N104	C	A	G	T	G	TC	T	T	C	G
Norway-1998	N105	C	GA	G	T	G	TC	CT	T	C	G
Norway-1998	N106	CT	G	A	C	G	C	C	T	C	G
Norway-1998	N107	C	GA		CT	G	TC		TA	C	G
Norway-1998	N108	C	GA	G	CT	G	TC	CT	T	CT	G
Norway-1998	N109	C	G	GA	CT	G	C	C	T	C	G
Norway-1998	N11	C	GA	GA	CT	G	C	CT	TA	CT	G
Norway-1998	N110	C		GA	C	GA	C		TA	C	G
Norway-1998	N111	C	G	G	CT	G	T	CT	T	C	G
Norway-1998	N112	C		GA		G	TC		T		G
Norway-1998	N113	CT	GA	GA	CT	G	TC	T	TA	C	G
Norway-1998	N114	C	GA	GA	CT	G	TC	CT	T	C	G
Norway-1998	N115	CT		GA	CT	G	TC	C	TA	CT	G
Norway-1998	N116	C		A		G	C	CT	TA	CT	G
Norway-1998	N117	C	GA	GA	C	G	C	CT	TA	C	G
Norway-1998	N118	CT	A	GA	CT	G	C	CT	T	C	G
Norway-1998	N119	C	G	G	CT	G	TC	C	T	CT	G
Norway-1998	N12	C		GA	T	G	TC	CT	TA	C	G
Norway-1998	N120	C	G		CT	G	C	CT	TA		G
Norway-1998	N121	C	G	G		G	TC	CT	T	CT	
Norway-1998	N122	CT	GA	GA	CT	G	TC	CT	TA	C	G
Norway-1998	N123	C	G	GA	C	G	C	CT	TA	CT	G

Norway-1998	N124	CT		G	C	A	T	CT	TA		
Norway-1998	N125	C	GA	GA	C		C	CT	T	CT	G
Norway-1998	N126	CT	G	G	CT	G	TC	C	T	CT	G
Norway-1998	N127	C	G		C	G	C	T	TA	C	
Norway-1998	N128	C	G	A	C	G	C	CT	A	CT	G
Norway-1998	N129	CT	G	GA	CT	G	TC	CT	T	C	
Norway-1998	N13	C	G	GA		G	C	CT	TA	CT	G
Norway-1998	N130	CT			CT	G	TC	CT	TA	C	
Norway-1998	N131	C	G	GA		G	TC	T	TA	C	G
Norway-1998	N132	C	GA	GA		G	TC	T	TA	C	G
Norway-1998	N133	C	G	GA	CT	G	TC	C	T	CT	G
Norway-1998	N134	C	A	GA	C	G	TC	C	T	CT	G
Norway-1998	N135	CT	GA	G		G	TC	T	T		G
Norway-1998	N136	C	G	GA		G	TC		TA	C	G
Norway-1998	N137	CT	G	G	CT	G	T	CT	T	T	G
Norway-1998	N138	C	GA	GA	C	G	TC	T	TA	T	G
Norway-1998	N139	T	GA	GA	C	GA	C	C	TA	CT	G
Norway-1998	N14										
Norway-1998	N140	C	GA	GA	CT	G	T	CT	T	C	G
Norway-1998	N141	C	G	A	C	G		CT	TA	CT	G
Norway-1998	N142	CT	GA	G	T	G	TC	CT	T	C	G
Norway-1998	N143	C	GA	A	C	G	C	CT	TA	CT	G
Norway-1998	N144	C	G	G	T	G	T	T	T	C	G
Norway-1998	N145	CT	G	A	C	G	TC	CT	TA	C	
Norway-1998	N146	C	A	G	T	G	TC	CT	T	CT	
Norway-1998	N147	C	G	A	C	G	TC	CT	T	C	
Norway-1998	N148	CT	GA	G	T	G	T	T	T	C	
Norway-1998	N149	CT	A	GA	CT	G	T	T	TA	C	
Norway-1998	N15										
Norway-1998	N150	C	GA	G	CT	G	TC	CT	T	CT	
Norway-1998	N151	C	GA	A	T	G	TC	CT	T	CT	G
Norway-1998	N152	CT	G	A	C	G	C	CT	TA	C	
Norway-1998	N153	C	A	G	CT	G	TC	CT	T	C	
Norway-1998	N154	C	G	GA		G	TC	CT	TA	C	
Norway-1998	N155	C	GA	G	CT	G	T	CT	T	C	
Norway-1998	N156	C	G	GA		G	T	T	TA	C	
Norway-1998	N157	C	GA	GA		G	TC	T	TA	CT	G
Norway-1998	N158	C	GA	A	C	G	C	C	T	CT	G
Norway-1998	N159	C	GA	GA	CT	G	TC	CT	T		G

Norway-1998	N16	C	GA	GA		G	TC	CT		C	G
Norway-1998	N160	C	G	GA		G	TC	T	TA	C	
Norway-1998	N17	C	GA	G	CT			C	T	CT	G
Norway-1998	N18	C	G	GA	CT	G	C	C	T	CT	G
Norway-1998	N19	T	GA	G	C	G	TC	C	T	CT	G
Norway-1998	N2	C		A		G	TC	CT	A		G
Norway-1998	N20	C	GA	GA	C	GA	TC	CT	TA	C	G
Norway-1998	N21	C	GA	G		G	TC	CT	T	CT	G
Norway-1998	N22	C	G	GA	CT	GA	T	CT	T	CT	G
Norway-1998	N23	T	A	GA	C		C	C	T	CT	
Norway-1998	N24										
Norway-1998	N25	C	A	G	T	G	TC	CT	T	C	
Norway-1998	N26	C	G	G	CT	G	TC	CT	T	C	G
Norway-1998	N27	C	G	GA	CT	G	TC	T	TA	C	G
Norway-1998	N28	CT	G	GA	T	GA	T	C	T		G
Norway-1998	N29	CT	G	GA	CT	G	C	CT	T	C	G
Norway-1998	N3	C	G		C	G	T	CT	T	C	G
Norway-1998	N30	C	G	G	C	GA	TC	C	T	C	G
Norway-1998	N31	C	GA	GA		G	C	CT	TA	C	G
Norway-1998	N32	C	G	GA	T	G	C	C	TA		G
Norway-1998	N33	CT	G	GA	C	G	TC	CT	TA	CT	G
Norway-1998	N34	CT	G	A	C	G	C	T	A	C	G
Norway-1998	N35	C	G	GA	C	G	C	T	TA	CT	G
Norway-1998	N36	C	G	GA	CT	G	C	C	TA	CT	G
Norway-1998	N37	C	GA	G	C	G	C	CT	TA	C	G
Norway-1998	N38	C	GA	A		G	C	CT	TA		G
Norway-1998	N39	C	G	G	T	G	T	T	T	C	G
Norway-1998	N4	C	G	G		G	TC	CT		CT	
Norway-1998	N40	CT	G	G	CT	GA	C	C	T	C	G
Norway-1998	N41	T	GA	G	T	G	T	T	T	C	G
Norway-1998	N42			GA	C	G		T	T	C	G
Norway-1998	N43			A		G	C	T	A	C	G
Norway-1998	N44	C	G	A	CT	G		CT	T	C	
Norway-1998	N45	C	GA	GA		G	C	CT	TA	C	G
Norway-1998	N46	C	GA	GA	C	G	C	C	T	C	G
Norway-1998	N47	CT	G		C	G	C	C	TA	CT	G
Norway-1998	N48	C	A	G	CT	G	TC	T	T	C	G
Norway-1998	N49	C	G		C	G	T	CT	TA	CT	G
Norway-1998	N5	C	G	GA	CT	G	TC	CT	TA	CT	

Norway-1998	N50	C	G	GA	C	GA	TC	T	TA	C	G
Norway-1998	N51	C	GA	GA	C		TC	C	T	C	G
Norway-1998	N52	C	G	G	CT	G	T	CT	T	C	G
Norway-1998	N53	CT	G	GA	C	G	TC	CT	TA	C	G
Norway-1998	N54	CT	GA	GA	C	G	TC	CT	T	C	G
Norway-1998	N55	T	A	G	T	G	T	T	T	C	G
Norway-1998	N56	CT	G	G	C	G	T	C	T	CT	G
Norway-1998	N57	C	G	GA	C	G	C	CT	TA	C	G
Norway-1998	N58	C	GA	GA	C	G	C	C	T	CT	G
Norway-1998	N59	T	A	GA	CT	G	T	CT	T	CT	G
Norway-1998	N6	T	G	A	C	G	C	T	A	C	
Norway-1998	N60	C	GA	GA		G	TC	T	TA	C	G
Norway-1998	N61	C		GA	CT	G	TC	CT	TA	C	G
Norway-1998	N62	C	A	G	T	G	TC	CT	T		
Norway-1998	N63	CT		GA		G		CT	TA	C	G
Norway-1998	N64	C	G	A	C	G	C	CT	TA	CT	
Norway-1998	N65	CT	G	A	C	G	C	CT	TA	C	G
Norway-1998	N66	C	G	GA	C	G	TC	CT	TA	C	G
Norway-1998	N67	CT	G	A	C	G	C	T	A	C	G
Norway-1998	N68	C	G	G	C	G	T	C	T	C	G
Norway-1998	N69	C	G	A	C	G	C	CT	TA	C	G
Norway-1998	N7	C	A	GA	T	G	T	T	T	C	
Norway-1998	N70	C	G	A		G	C	CT	TA	CT	G
Norway-1998	N71	C	G	GA		G	TC	T	TA	C	
Norway-1998	N72		G	GA		G	C	T	TA	C	G
Norway-1998	N73	C	G	G	C	G	TC	C	T	C	G
Norway-1998	N74	C	GA	G	CT	G	TC	C	T	C	G
Norway-1998	N75	CT	GA	A	C	G	C	CT	TA	C	
Norway-1998	N76	C	G	GA	C	G	TC	C	T	CT	G
Norway-1998	N77	C	G	A	C	G	TC	C	T	C	
Norway-1998	N78	C	G	GA	C	G	TC	CT	TA	C	G
Norway-1998	N79	CT	G	GA	C	G	T		T	C	G
Norway-1998	N8	C					TC	CT	TA	CT	G
Norway-1998	N80	C	GA	GA	CT	G	C	CT	T		G
Norway-1998	N81	C		GA	CT	A	T		T	C	
Norway-1998	N82	C	G	GA		G	TC	T	TA	C	
Norway-1998	N83	C	GA	G	CT	G	TC	CT	T	CT	
Norway-1998	N84	C		GA		G	C	CT	T	C	G
Norway-1998	N85		GA		C	G	TC	CT	TA	C	G

Norway-1998	N86	C	A		CT	G		T	T		
Norway-1998	N87			GA	C	G		T	TA	C	
Norway-1998	N88		GA	GA		G	TC	CT	TA	C	G
Norway-1998	N89	CT	GA	G	CT	G	TC	CT	T	C	G
Norway-1998	N9	CT	G	GA	C	G	C	T	TA	C	G
Norway-1998	N90	C	GA	GA	C	G	C	CT	TA	C	G
Norway-1998	N91	C	A	GA	C	G	T	C	T	CT	G
Norway-1998	N92	C	GA	GA	CT		TC	CT	T	CT	G
Norway-1998	N93	C	G	A		G	C	CT	TA	C	G
Norway-1998	N94	C	G	A	C	G	TC	CT	TA	C	G
Norway-1998	N95	CT	G	G	C	G	C			CT	G
Norway-1998	N96	CT	G	GA		G	C	CT	TA	C	
Norway-1998	N97	C	G	A	C	G	TC	T	TA	CT	G
Norway-1998	N98	C	GA	GA	CT	G	TC	CT	T	C	G
Norway-1998	N99	C	GA	A	C	G	C	T	A	C	
Scotland-2002_a	S1	C	G	A	T	G	C	C	TA	CT	G
Scotland-2002_a	S10	C	G	A	CT	G	TC	CT	TA	C	G
Scotland-2002_a	S11	CT	G	A	CT	G	TC	C	TA	C	G
Scotland-2002_a	S12	CT		A	CT	G	TC	C	TA	C	G
Scotland-2002_a	S13	C	G	A	CT	G	C	CT	A	C	G
Scotland-2002_a	S14	C	G	A	T	G	C	C	A	C	G
Scotland-2002_a	S15	CT	G	A	T	G	TC	C	TA	CT	G
Scotland-2002_a	S16	C	G	A	T	G	TC	C	TA	C	G
Scotland-2002_a	S17	C	G	A	T	G	C	C	A	C	G
Scotland-2002_a	S18	C	G	A	CT	G	C	C	TA	C	G
Scotland-2002_a	S19	C	G	A	CT	G	TC	C		C	G
Scotland-2002_a	S2	C	G	GA	CT	GA	C	C	T	C	
Scotland-2002_a	S20	C	G	A	T	G	C	C	A	C	G
Scotland-2002_a	S21	C	A	GA	CT	G	T	CT	TA	CT	G
Scotland-2002_a	S22	C	GA	GA	CT	G	TC	C	TA	CT	G
Scotland-2002_a	S23	C	G	A	CT	G	TC	C	A	C	G
Scotland-2002_a	S24	C	G	A	T	G	TC	C	T	C	G
Scotland-2002_a	S25	C	G	A	CT	G	C	C	TA	C	G
Scotland-2002_a	S26	C	GA	A	T	G	C	C	A	C	G
Scotland-2002_a	S27	C	G	A	T	GA	C	C	T	C	G
Scotland-2002_a	S28	C	GA	GA	T	G	TC	CT	TA	CT	G
Scotland-2002_a	S29	T	G	GA	T	G	C	C	T	C	G
Scotland-2002_a	S3	C			T	G	C	C	TA	C	G
Scotland-2002_a	S30	C	GA	A	CT	G	TC	C	A	C	G

Scotland-2002_a	S31	C	GA	GA	CT	G	C	CT	TA	CT	G
Scotland-2002_a	S32	C	G	A	T	G	C	C	TA	C	G
Scotland-2002_a	S33	C	G	A	C	G	C	C	T	C	G
Scotland-2002_a	S34	C	G	A	CT	G	TC	C		CT	
Scotland-2002_a	S35	C	G	GA	T	G	C	C	TA	CT	G
Scotland-2002_a	S36	C	G	A	T	G	C	C	TA	C	G
Scotland-2002_a	S37	C	GA	GA	CT	G	C	C	T	C	G
Scotland-2002_a	S38	C	GA	GA	T	G	T	C	T	T	G
Scotland-2002_a	S39	C	GA	A	T	GA	C	C	T	C	G
Scotland-2002_a	S4	C	GA	A	CT	G	C	C	TA	C	G
Scotland-2002_a	S40	C	G	GA	CT	G	TC	C	TA	CT	
Scotland-2002_a	S41	CT	GA	A	T	G	C	CT	TA	C	G
Scotland-2002_a	S42	C	GA	A	CT	G	C	C	TA	CT	
Scotland-2002_a	S43	C	GA	G	C	G	TC	C	T	CT	G
Scotland-2002_a	S44	C	G	A	CT	G			A	C	G
Scotland-2002_a	S45	C	G	A	CT	G	TC	C	TA	CT	G
Scotland-2002_a	S46	C	G	A	T	G	C	C	A	C	A
Scotland-2002_a	S47	C	G	A	CT	G	TC	C	TA	CT	G
Scotland-2002_a	S48	C	G	A	CT	G	TC	C	TA	CT	G
Scotland-2002_a	S49	C	G	GA	CT	GA	C	CT	TA	C	G
Scotland-2002_a	S5	CT	G	A	CT	G	C	C	T	C	G
Scotland-2002_a	S50	CT	G	A	CT	G	TC	C	TA	CT	G
Scotland-2002_a	S51	CT	GA	A	T	G	C	C	TA	CT	G
Scotland-2002_a	S52	C	GA	GA	T	G	TC	CT	T	C	G
Scotland-2002_a	S53	C	G	A	T	G	C	C	T	C	G
Scotland-2002_a	S54	C	GA	GA	T	GA	C	C	TA	CT	G
Scotland-2002_a	S55	C	G	A	CT	G	TC	C		C	G
Scotland-2002_a	S56	C	G	A	C	G	TC	CT	T	CT	G
Scotland-2002_a	S57	C		GA	T	G	TC	C	TA	CT	G
Scotland-2002_a	S58	C	G	A	T	G	C	C	T	CT	G
Scotland-2002_a	S59	C		A	CT	G	TC	C	TA	CT	G
Scotland-2002_a	S6	C	G	A	T	G		C		C	
Scotland-2002_a	S60	C	G	GA	CT	G	C	CT	TA	C	G
Scotland-2002_a	S7	C	G	A		G	C	C	A	C	G
Scotland-2002_a	S8	CT	G	A	CT	G	TC	C	TA	C	G
Scotland-2002_a	S9	C	G	A	T	G	C	C	TA	C	G
